# The comprehensive SARS-CoV-2 ‘hijackome’ knowledge base

**DOI:** 10.1038/s41421-024-00748-y

**Published:** 2024-12-09

**Authors:** Sini Huuskonen, Xiaonan Liu, Ina Pöhner, Taras Redchuk, Kari Salokas, Rickard Lundberg, Sari Maljanen, Milja Belik, Arttu Reinholm, Pekka Kolehmainen, Antti Tuhkala, Garima Tripathi, Pia Laine, Sergei Belanov, Petri Auvinen, Maria Vartiainen, Salla Keskitalo, Pamela Österlund, Larissa Laine, Antti Poso, Ilkka Julkunen, Laura Kakkola, Markku Varjosalo

**Affiliations:** 1 Institute of Biotechnology, Helsinki Institute of Life Science HiLIFE, University of Helsinki, Helsinki, Finland; 2 School of Pharmacy, University of Eastern Finland, Kuopio, Finland; 3 Institute of Biomedicine, University of Turku, Turku, Finland; 4 Finnish Institute for Health and Welfare, THL, Helsinki, Finland; 5 Clinical Microbiology, Turku University Hospital, Turku, Finland; 6 InFlames Research Flagship Center, University of Turku, Turku, Finland

**Keywords:** Proteomics, Mechanisms of disease

## Abstract

The continuous evolution of SARS-CoV-2 has led to the emergence of several variants of concern (VOCs) that significantly affect global health. This study aims to investigate how these VOCs affect host cells at proteome level to better understand the mechanisms of disease. To achieve this, we first analyzed the (phospho)proteome changes of host cells infected with Alpha, Beta, Delta, and Omicron BA.1 and BA.5 variants over time frames extending from 1 to 36 h post infection. Our results revealed distinct temporal patterns of protein expression across the VOCs, with notable differences in the (phospho)proteome dynamics that suggest variant-specific adaptations. Specifically, we observed enhanced expression and activation of key components within crucial cellular pathways such as the RHO GTPase cycle, RNA splicing, and endoplasmic reticulum-associated degradation (ERAD)-related processes. We further utilized proximity biotinylation mass spectrometry (BioID-MS) to investigate how specific mutation of these VOCs influence viral–host protein interactions. Our comprehensive interactomics dataset uncovers distinct interaction profiles for each variant, illustrating how specific mutations can change viral protein functionality. Overall, our extensive analysis provides a detailed proteomic profile of host cells for each variant, offering valuable insights into how specific mutations may influence viral protein functionality and impact therapeutic target identification. These insights are crucial for the potential use and design of new antiviral substances, aiming to enhance the efficacy of treatments against evolving SARS-CoV-2 variants.

## Introduction

The emergence of the severe acute respiratory syndrome coronavirus 2 (SARS-CoV-2) in late 2019 led to the ongoing COVID-19 pandemic, which has had profound global health and socio-economic consequences^1^. SARS-CoV-2 continuously evolves, giving rise to numerous variants of concern (VOCs) with distinct genetic and phenotypic characteristics^2,3^. Phylogenetic analyses have revealed that these VOCs diverged autonomously from the ancestral lineage of wave 1 (W1) viruses. Among them, Alpha (B.1.1.7), Delta (1.617.2), and Omicron (BA.1 and BA.5) have spread worldwide, while Beta (B.1.351) and Gamma (P.1) have largely remained geographically confined. The Omicron variant with its extensively mutated spike (S) protein signified the most dramatic antigenic shift to date, evading the adaptive immunity fostered by vaccinations and prior infections^4–7^. Subsequent Omicron subvariants BA.4 and BA.5 swiftly superseded Omicron BA.1 and BA.2, marking Omicron as the first VOC to spawn globally as dominant sub-lineages^5,8^. More recently, Omicron variants XBB1.5, BA2.86 and JN.1 have been prevalent in different countries.

**Fig. 1:**
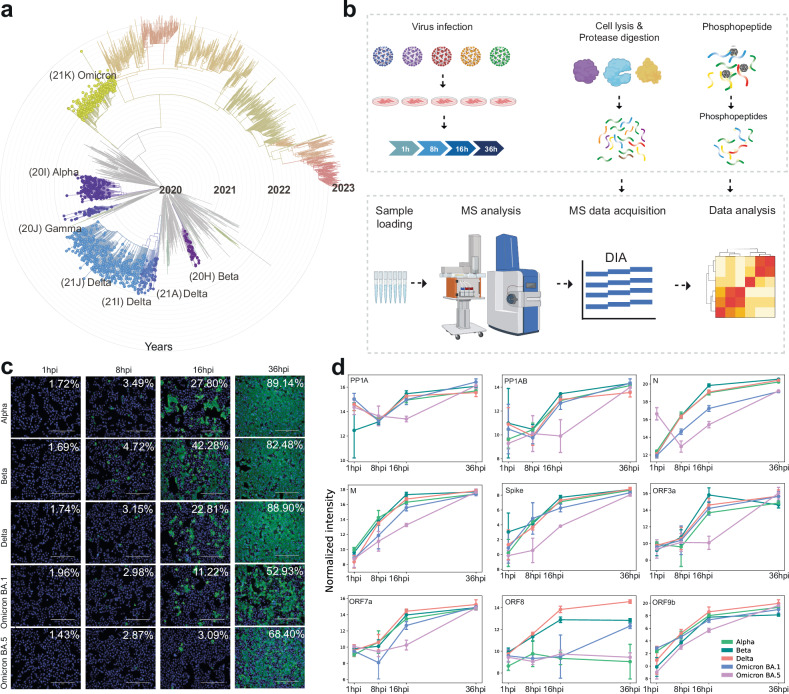
Overview of SARS-CoV-2 VOCs and the experimental approach to study the host–pathogen interactions. **a** The epidemiological and evolutionary tree of the SARS-CoV-2 variants (modified from Nextstrain.org), illustrating the emergence of the key SARS-CoV-2 VOCs (Alpha, Beta, Gamma, Delta, and Omicron). **b** Scheme of the experimental workflow: samples were collected at various time points post infection (1 hpi, 8 hpi, 16 hpi, and 36 hpi), followed by global quantitative proteomics and phosphoproteomics analyses using DIA-PASEF. **c** Infection progression was monitored by detecting the SARS-CoV-2 N protein through immunofluorescence microscopy. **d** Normalized log_2_-transformed intensity of SARS-CoV-2 proteins expressed during infection in the VeroE6-H10 cells. The error bars represent one standard deviation, *n* = 3 biological replicates.

VOCs are thought to have arisen due to evolutionary pressures to adapt to human hosts and to evade both innate and adaptive immune responses, thereby enhancing human-to-human transmission^9,10^. The S protein of SARS-CoV-2 facilitates cellular entry via the angiotensin-converting enzyme 2 (ACE2) receptor and has emerged as the most altered protein across VOCs^2,11^. However, VOCs have also acquired a multitude of non-synonymous mutations in other proteins, including non-structural, structural, and accessory proteins, such as NSP3, NSP6, NSP9, NSP12, NSP13, nucleocapsid (N), membrane (M), envelope (E), ORF3a, ORF6, ORF7a, ORF7b, ORF8a, and ORF9b^3,12^. The spectrum of mutations spans widely; VOCs from Alpha to Delta variants possess 20–29 non-synonymous consensus mutations, while Omicron BA.1 possesses more than 60 mutations, predominantly in the S protein (Fig. 1a; Supplementary Table S1). Additionally, each VOC bears 4–10 non-coding mutations.

A multifaceted approach integrating genomic and proteomic analyses is indispensable for studying SARS-CoV-2 variants within host cells. Techniques such as next-generation sequencing enable the decoding of the complete viral genome and the identification of specific mutations, thus aiding in tracking the emergence and dissemination of variants. Concurrently, proteomics allows the identification of the virus-encoded proteins that underpin viral functions and host interactions. It sheds light on how variants modify protein expression, structure, and function, thereby influencing disease progression and treatment efficacy.

While extensive proteomics research has been conducted on SARS-CoV-2 evolution, many studies have focused predominantly on specific proteins or a limited selection of viral protein alterations of one variant. For instance, previous studies have concentrated on the Wuhan strain and confined the analysis to a single time point, potentially overlooking the full spectrum of responses within the host cells. Additionally, some studies, whilst offering valuable insights, have solely focused on major open reading frames (ORFs) of SARS-CoV-2, possibly omitting the effects on individual NSPs and specific mutations altering virus functions^13–15^.

Our current study expands the proteomics exploration, delving into the complete ‘hijackome’ during the time course of infection, especially at later time points than previously observed, with five prevalent SARS-CoV-2 VOCs. Utilizing VeroE6-H10 cell cultures infected with Finland-derived Alpha, Beta, Delta, and Omicron BA.1 and BA.5 VOCs, our analysis spans 1–36 h post infection (hpi) to trace the virus’s trajectory during the infection. We utilized cutting-edge phosphoproteomics to examine phosphorylation dynamics throughout the course of the infection, and to discover the effects of mutations of SARS-CoV-2 VOC proteins. We further introduced the VOC-specific mutations of interests into 12 viral proteins and employed BioID-MS to decipher mutation-related alterations in protein interactions. Our approach yields profound insights into the functional consequences of these mutations, which affect vital cellular pathways, such as RHO GTPases, MAPK-, EGFR-signaling, and endoplasmic reticulum (ER)-associated processes. The identification of the role of these pathways in the response to infection with SARS-CoV-2 VOCs is instrumental in understanding the virus’s symptomatology, replication mechanisms, and prospective therapeutic avenues.

## Results

### SARS-CoV-2 variants efficiently infect the host cells with differing infection dynamics

Our primary objective was to systematically and comprehensively investigate the infection landscape of the SARS-CoV-2 VOCs to produce a complete map of the SARS-CoV-2–host cell interactions or ‘hijackome’. SARS-CoV-2 is a +ssRNA virus of ~29.9 kb in size, featuring a complex genetic structure comprising *ORF1a* and *ORF1b* that encode polyproteins (ORF1a and ORF1b, respectively), as well as other regions responsible for accessory and structural proteins, such as S, E, M, and N proteins, that are vital for viral particle formation and RNA interaction within the viral core^16^. We included the VOCs Alpha (B.1.1.7), Beta (B.1.351), Delta (B.1.617.2) and, Omicron variants BA.1 and BA.5 (Fig. 1a) which are known to have accumulated multiple mutations leading to structural and functional changes in viral proteins^3,17^.

To study the functional effects of SARS-CoV-2 infection and possible differences between the VOCs, we quantified the differences in the total and phosphorylated proteomes (phosphoproteomes) at different time points of infection (1 hpi, 8 hpi, 16 hpi, 36 hpi) in VeroE6-TMPRSS2-H10 cells using a Data-Independent Acquisition combined with Parallel Accumulation Serial Fragmentation (DIA-PASEF) quantitative proteomics analysis pipeline (Fig. 1b). We assessed infection progression with fluorescence microscopy (Fig. 1c), and the viral protein levels with DIA-PASEF (Fig. 1d). We observed that in cells infected with Omicron BA.1, and especially with BA.5, viral protein expression progressed at a slower rate at the early time points (1 hpi, 8 hpi, 16 hpi) compared to Alpha, Beta, and Delta variants. Regardless of the somewhat different virus titers in inoculation for Alpha and Delta variants, the slower kinetics in Omicron infection are clearly evident compared to the Beta variant infected with equal virus titer. However, BA.1 and BA.5 infections rapidly reached the levels of the other variants, and the viral protein levels at the 36 hpi time point were comparable to those of Alpha, Beta, and Delta VOCs (Fig. 1d; Supplementary Table S2). This finding is in agreement with previous reports of Omicron infection dynamics^18,19^. Additionally, we noted that the Alpha variant exhibited very low levels of ORF8a expression. This is likely explained by the ORF8a Q27stop mutation, identified in Alpha variant, either initiating nonsense-mediated mRNA decay or resulting in an unstable protein (Fig. 1d). Interestingly, we detected similarly low levels of ORF8a expression in the BA.5-infected cells. This validates the sequencing finding that BA.5 contains a mutation (C27889T) in the middle of the ORF8a transcriptional regulatory sequences (TRS), that is predicted to reduce or ablate ORF8a expression^20^. The lack of ORF8a expression is most likely beneficial for the virus, as the ORF8a induces monocytic pro-inflammatory cytokine expression via NLPR3 inflammasome pathways^21^.

### Cells infected with SARS-CoV-2 variants exhibit similar host cell responses at the protein level

**Fig. 2:**
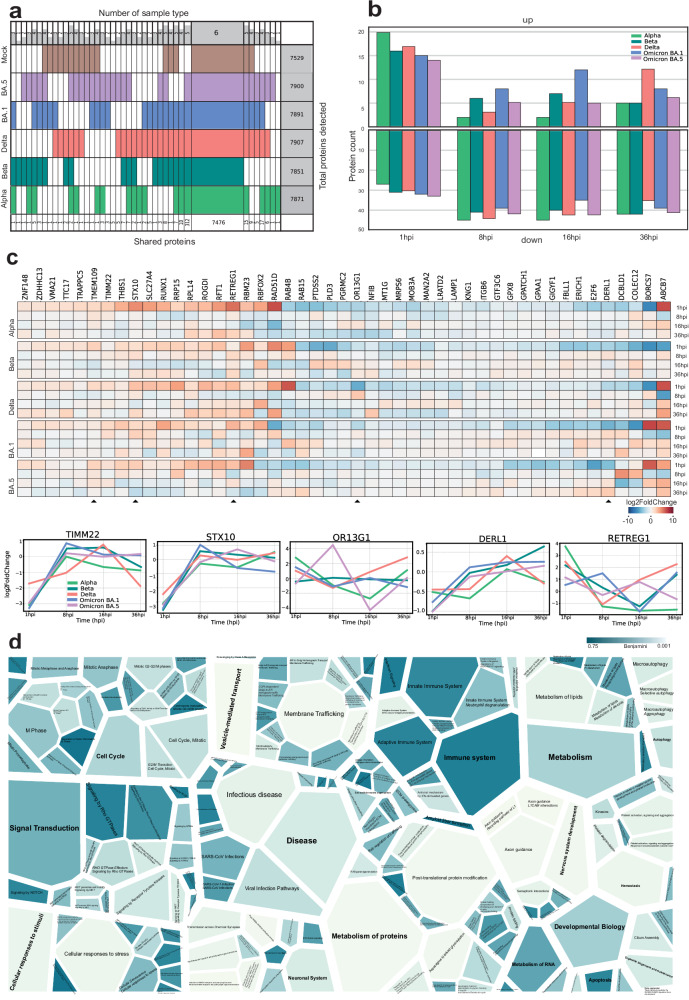
Comprehensive quantitative proteomics analysis of total proteome changes of the host cells upon infection with the SARS-CoV-2 variants. **a** Super Venn representation of the total number of proteins quantified in each sample group from all time points. Each row corresponds to an individual variant. Key: Alpha, light green; Beta, dark green; Delta, coral; Omicron BA.1, blue; Omicron BA.5, lilac; mock, brown. The numbers at the bottom of each column indicate the total number of proteins shared within the group, while the numbers on top indicate the number of sample groups within the shared set. Column on the right presents the total identifications within the group. **b** Column graph comparing total proteome data to the mock sample at each time point, illustrating the number of proteins exhibiting either upregulation or downregulation relative to the prior sample time point. **c** Variant-specific filtering analysis to identify SARS-CoV-2 variant-specific differences. In this analysis, mock samples were used as a baseline, with data filtering and normalization highlighting statistically significant alterations. Proteins with significant up- or downregulation compared to mock samples were selected, identifying 47 proteins with notable changes (log₂FC) across time points (1 hpi, 8 hpi, 16 hpi, and 36 hpi) in VOCs. Additionally, proteins with consistent expression changes across all sampled time points and variants are represented in a heatmap arranged alphabetically. Five selected proteins (TIM22, STX10, OR13G1, DERL1, and RETREG1) are shown in an alternative format to display their log₂FC. **d** Voronoi graph representing the complete SARS-CoV-2 ‘hijackome’ of host cell functions on 36 hpi, analyzed using enrichment of Reactome pathways of virus-infected cells compared to the mock cells. Proteins with significant up- or downregulation (Benjamini-Hochberg corrected *P* < 0.05, log_2_FC) in any variant-infected cells relative to mock were included in the Reactome enrichment analysis, with color gradients illustrating overrepresentation based on adjusted *P* values (Benjamini).

Next, we examined the virus-host protein responses induced by SARS-CoV-2 VOCs on the VeroE6-TMPRSS2-H10 host cell proteome using the DIA-PASEF analysis pipeline (Fig. 1b). We detected and quantified similar numbers of host proteins in all samples, ranging from 7529 quantified proteins in the samples from mock-infected cells, to 7851–7900 proteins in the samples from SARS-CoV-2 VOC-infected cells (Fig. 2a). Of these, 312 proteins were found to be unique for the SARS-CoV-2-infected cells (Supplementary Table S2). This larger number of proteins quantified in samples from infected cells reflects the changes induced by viral infection in host protein expression. We used Reactome pathway analysis to identify the processes to which these 312 proteins were linked (Supplementary Table S2). The analysis identified five statistically significantly enriched (Benjamini adjusted *P* < 0.001) pathways; ‘Translation of Accessory Proteins’ (*P* = 1.69E–05), ‘RUNX3 regulated NOTCH signaling’ (*P* = 3.97E–04), ‘Virion Assembly and Release’ (*P* = 4.67E–04), ‘Regulation of beta-cell development’ (*P* = 6.06E–04), and ‘Regulation of gene expression in beta cells’ (*P* = 9.43E–04).

### Temporal and variant-specific host protein expression dynamics during SARS-CoV-2 infection

The effects of a viral infection on the transcriptional and translational processes of the host cells occur dynamically and can be observed at different stages post infection. To dissect the temporal- and VOC-specific variations seen in host cells, we investigated the number of proteins upregulated or downregulated at different time points post infection (1 hpi, 8 hpi, 16 hpi, and 36 hpi) across VOCs. This analysis shed light on the molecular responses induced by various SARS-CoV-2 variants over time, particularly on 36 hpi (Fig. 2b; Supplementary Table S2) offering insights into how each variant may impact the host cellular machinery. Furthermore, to determine SARS-CoV-2 variant-specific differences between VOCs, we compared the proteome data from virus-infected cells to mock-infected cells. We identified 47 proteins that exhibited significant changes when comparing the proteomes of VOCs to mock samples across multiple time points (Fig. 2c; Supplementary Table S2). Each of these proteins appeared to be significantly (log_2_Fold Change (FC)) changed at least once (in a time point) in one of the VOCs when compared to the mock samples. This visualization underscores the proteins that consistently demonstrate significant abundance changes, shedding light on their potential roles in the host response to SARS-CoV-2 variants over time.

Interestingly, the majority of these differentially expressed proteins have previously been linked to processes related to infections by viruses or other pathogens. Of the differentially expressed proteins, TIMM22, a protein responsible for mitochondrial protein import^22,23^, showed a pattern of downregulation at 1 hpi, which transitioned to upregulation at around 8–16 hpi across variants, suggesting that the SARS-CoV-2 infection impacts on mitochondrial processes. STX10, a SNARE protein involved in vesicular transport processes during infection^24^, exhibited diverse expression patterns in response to SARS-CoV-2 variants. In most variants, including Alpha, Beta, Delta, and Omicron BA.1, STX10 showed initial downregulation at 1 hpi followed by upregulation during the early and middle stages of infection. However, with the Omicron BA.5 variant, STX10 appeared to be more downregulated as the infection progressed, with its expression decreasing in later stages.

OR13G1, an olfactory receptor protein, displayed strong upregulation in the later stages of Alpha and Delta variant infections, contrasting with consistent protein expression levels throughout the infection by Beta and BA.1 variants, while exhibiting early upregulation followed by later downregulation in the case of BA.5. This dynamic modulation suggests a potential link between OR13G1 and SARS-CoV-2 symptoms, particularly the loss of smell^25^.

Two proteins with roles in the ER were expressed in a variant-specific manner. DERL1 is a critical component of the ER-associated degradation (ERAD) pathway and responsible for eliminating misfolded luminal proteins^26,27^. It displayed variant-specific behavior, with the Beta variant notably upregulating DERL1 throughout infection, and similar trends seen in variants BA.1 and BA.5, while Alpha and Delta seemingly going up in middle stages of the infection and returning to their original levels. RETREG1 is an ER-anchored autophagy regulator known to be influenced by the SARS-CoV-2 virus’s promotion of reticulophagy^28^. This process triggers ER stress and inflammatory responses, contributing to viral infection dynamics. In the Alpha and BA.5 variants, RETREG1 expression remains consistently downregulated and continues to decrease over time, while in other variants there is a resurgence of rising expression in the middle or later stages of infection. Furthermore, the ERAD pathway, critical for degrading misfolded viral proteins, is activated in response to SARS-CoV-2 infection, in an attempt to manage increased protein load and restore ER homeostasis in the host cell^29,30^.

### Comprehensive analysis of host cell signaling pathways and protein regulation during infection at 36 hpi

Finally, to obtain a more comprehensive view of the proteins and host cell signaling pathways affected during infection, we employed Reactome pathway analysis for all significantly up- or downregulated proteins at fully developed infection at 36 hpi (Benjamini adjusted *P* < 0.05, log_2_FC ± 1 using the Database for Annotation, Visualization, and Integrated Discovery (DAVID) bioinformatics tools), and visualized the enriched Reactome signaling pathways (Fig. 2d; Supplementary Table S2). Several parent terms and their subterms were significantly enriched, such as the term ‘Vesicle-mediated transport’ (adjusted *P* = 7.0E–4 and 43 proteins) and its subterm ‘Membrane Trafficking’ (*P* = 6.2E–3 and 38 proteins), the term ‘Metabolism of proteins’ (*P* = 6.2E–3 and 90 proteins) and its subterm ‘Post-translational protein modification’ (*P* = 8.6E–3 and 68 proteins), the term ‘Nervous System development’ (*P* = 1.4E–2 and 34 proteins) and its subterm ‘Axon guidance’ (*P* = 1.4E–2 and 33 proteins), and the term ‘Cellular responses to stimuli’ (*P* = 2.7E–2 and 42 proteins) with its subterm ‘Cellular responses to stress’ (*P* = 2.1E–2 and 42 proteins). In addition, ‘Infectious disease’ (*P* = 4.4E–2 and 48 proteins) and ‘RHO GTPases Activate Formins signaling by Rho GTPases’ (*P* = 8.1E–2 and 12 proteins) were enriched. Overall, we identified several significantly up- or downregulated proteins associated with key biological processes and signaling pathways.

### Phosphoproteomics analysis reveals pathways activated by SARS-CoV-2 infection

Since protein phosphorylation is one of the key mechanisms in the regulation of cellular signaling pathways, analyzing phosphorylation events provides critical insights into how viruses affect host cell functions and processes. Therefore, we performed phosphopeptide enrichment, coupled with quantitative mass spectrometry (MS), on the samples obtained from SARS-CoV-2 VOC-infected cells. Classifying these phosphorylation events enabled us to deduce the activity of different host cell signaling pathways^31^, which provides insights into the molecular mechanisms of infection and can identify potential new therapeutic targets^32,33^.

**Fig. 3:**
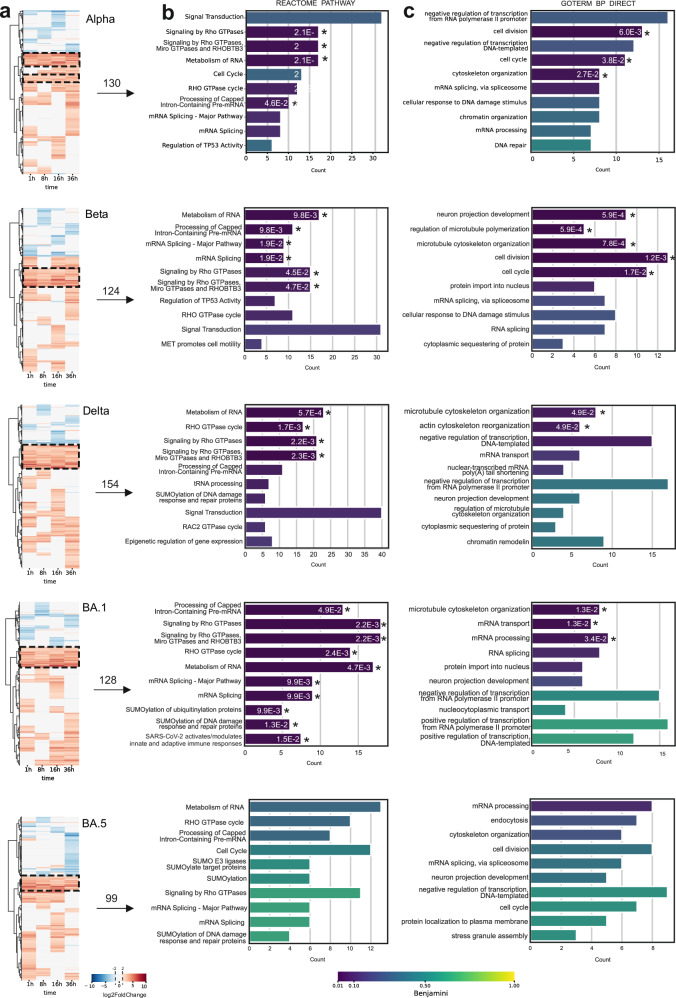
Time-course phosphoproteome analysis of SARS-CoV-2 variant infection. **a** Cluster plot illustrating the temporal dynamics of protein phosphorylation across different time points during SARS-CoV-2 variant infections. A comprehensive comparison was conducted between variants and mock cells to identify the variant-specific upregulation (red) or downregulation (blue). The numbers above the arrows represent the unique proteins detected in each boxed upregulated cluster. **b**, **c** Selected clusters of consistently upregulated phosphorylation events were analyzed for Reactome pathway enrichment (**b**) and GO Biological Process enrichment (**c**) using the DAVID functional annotation tool. The top 10 enriched pathways and processes, ranked by count, are shown with their respective adjusted *P*-values (Benjamini). An asterisk adjacent to each bar indicates a statistically significant enrichment threshold (*P* < 0.05), with the corresponding adjusted *P*-value.

We analyzed the phosphoproteomes of different SARS-CoV-2 VOC-infected cell proteins at various time points and identified a total of 488,883 peptides from the entire dataset, encompassing 2500 unique phosphosites from 1395 unique proteins (Supplementary Table S3). To visualize the phosphoproteomal changes during the infection with different SARS-CoV-2 variants, we hierarchically clustered each of the quantified phosphoproteomes (Fig. 3a). The heatmaps display each quantified phosphosite as log_2_FC compared to the mock. Only phosphosites with log_2_FC of ≤ –2 and ≥ 2 are shown. All variants displayed clear clusters of phosphosites consistently upregulated through all time points (dashed box). We did not detect any clear clusters of consistently downregulated phosphosites. The number of unique proteins containing upregulated phosphosites ranged from 99 (Omicron BA.5 variant) to 154 (Delta variant).

Subsequently, we performed Reactome (Fig. 3b) and Gene Ontology (GO) Biological Process term enrichment analysis on the phosphoproteins from these clusters from all time points (Fig. 3c; Supplementary Table S3). Omicron BA.5 infection did not result in statistically enriched terms as observed in the other variants, but showed similar trends. Focusing on the Reactome pathways, a clear and consistent trend of enrichment (Benjamini adjusted *P* < 0.05) in the RHO GTPase-related terms (‘Signaling by Rho GTPases’, ‘Signaling by Rho GTPases, Miro GTPases and RHOBTB3’, and ‘Rho GTPase cycle’) was observed (Fig. 3b). The Alpha, Beta, Delta and BA.1 variant-infected samples contained ≥ 15 proteins linked to those terms. Additionally, mRNA related-terms ‘Metabolism of RNA’, ‘Processing of Capped Intron-containing Pre-mRNA’, and ‘RNA Splicing—Major Pathway’, were significantly enriched with ~10 proteins in each variant-infected cells, except in that of BA.5. Similar processes were detected with GO Biological Process enrichment analysis, and additionally we found cell cycle-related terms (‘cell division’ and ‘cell cycle’) to be enriched upon infection with Alpha and Beta variants only (adjusted *P* < 0.05). Interestingly, Beta variant-infected samples also displayed enrichment of the ‘neuron projection development’ term, which was not detected in other variant-infected cells.

### Variants differentially affect the phosphorylation of various host cell kinases

**Fig. 4:**
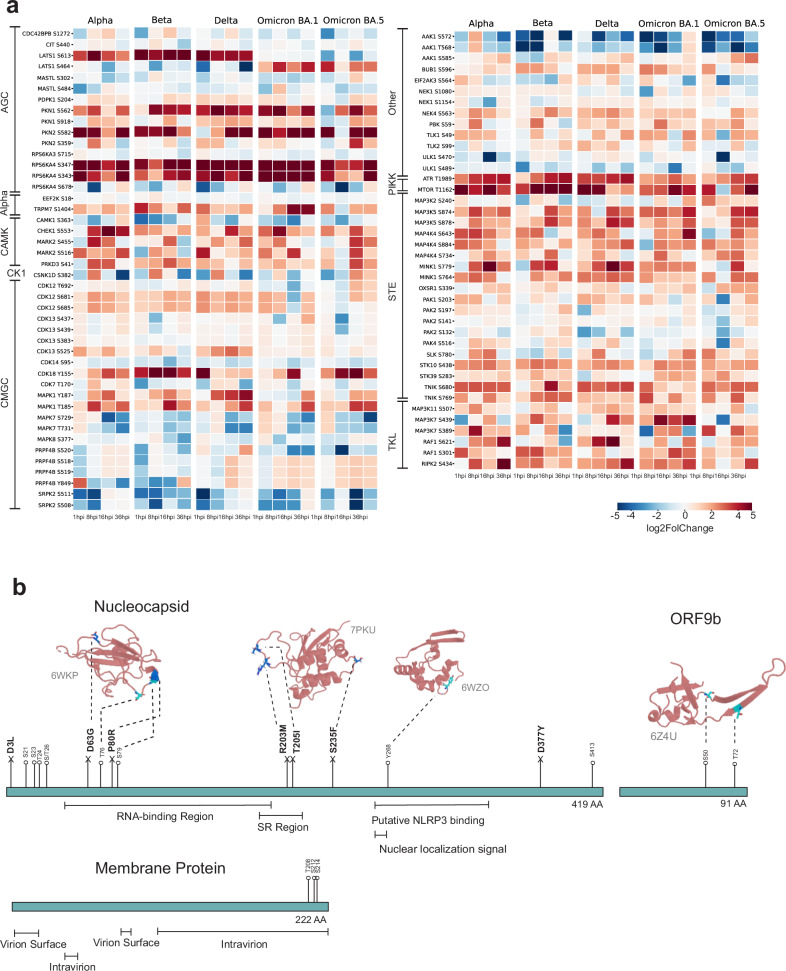
Protein kinase phosphorylation during SARS-CoV-2 infection. **a** Identified protein kinase phosphorylation events (phosphosites) detected and compared to mock. Red represents upregulation and blue indicates downregulation. **b** Protein phosphorylation events detected in the SARS-CoV-2 N, ORF9b, and M proteins, with their position shown in the corresponding protein sequence in cyan, and the ORF-specific mutations highlighted in marine.

As regulators of cellular signaling, protein kinases themselves are known to be activated by phosphorylation and to have key roles in viral infection^32,34^. Therefore, we examined the SARS-CoV-2 VOC infection-induced phosphorylation changes in the host kinome. Our analysis revealed distinct variant- and time point-specific disparities in protein kinase phosphorylation between VOC-infected and the mock cells (Fig. 4a; Supplementary Table S4). We detected several clusters in the host kinome, of which three showed clear upregulation and two downregulation on certain phosphosites in specific kinases.

Phosphorylations of several members of the AGC kinase family, named after the protein kinase A, G, and C families (PKA, PKC, PKG^35^) were upregulated during infection. Strong upregulation in kinase phosphosites was detected in kinases LATS1 (S464 and S613), PKN1 (S562), PKN2 (S582), and RPS6KA4 (S343 and S347). Interestingly, the predominantly upregulated LATS1 phosphosite differed between the variants, with S613 being phosphorylated upon infection with Alpha, Beta, and Delta variants, and S464 upon Omicron BA.1 or BA.5 variant infection. Phosphorylation of S464 by NUAK1 and NUAK2 is known to lead to decreased LATS1 protein levels. LATS1 is involved in regulation of cellular senescence and cellular ploidy^36^.

We detected robust phosphorylation of kinases PKN1 and PKN2 upon infection with all variants. PKN1 and PKN2 kinase activity is activated upon binding to Rho proteins (RHOA, RHOB and RAC1), connecting RHO GTPAse signaling pathway detected in enrichment result to these phosphorylation results^37,38^. PKN1 and PKN2 kinase activities are also regulated by caspase-3 (CASP3) cleavage during apoptosis^39,40^. The S562 phosphosite in PKN1 detected in our phosphorylation results is proximal to the caspase-3 cleavage site, D558 and D560, and thus possibly regulates the cleavage. We also detected strong upregulation of phosphorylation of kinase RPS6KA4 on phosphosites S343 and S347. The phosphosite S343 is known to be phosphorylated by the upstream kinases MAPK1 and MAPK3, and it is also autophosphorylated, a phenomenon essential for its catalytic activity^41^.

Phosphorylations of the PIKK kinases ATR (T1989) and MTOR (T1162) were upregulated by infection with all variants. The PIKK kinases are known to participate in V(D)J recombination, meiotic division, chromosome maintenance, sensing DNA damage and DNA damage repair, and cell cycle regulation^42^. The phosphorylation of ATR T1989 autophosphorylation site is required for its kinase activity^43^.

Various STE kinases showed increased phosphorylation in response to infection with all variants and at most time points post infection. These include MAPK (Mitogen-Activated Protein Kinase) kinases; MAP3K2 (S240), MAP3K5 (S874 and S878), MAP4K4 (S643, S734, and S884); and STK10 (S438), STK39 (S283), and TNIK (S680 and S769). The role of MAPKs in viral infection signaling has been suggested also for coronaviruses^44^.

It is worth mentioning that we also detected downregulation of phosphorylation of several CMGC kinase family kinases. For example, phosphorylation of PRPF4B (Y849) is highly downregulated upon infection by Alpha and Beta, but not by Delta, Omicron BA.1, and Omicron BA.5 variants. Phosphorylation of SRPK2 (S511 and S508) is upregulated in later stages of viral infection, especially in infection with Alpha, Delta and Omicron BA.1 variants. Both of these kinases are involved in pre-mRNA-related processes such as RNA splicing^45^. These findings emphasize the specificity of phosphorylation events associated with distinct SARS-CoV-2 variants.

We also detected extensive downregulation of AAK1 phosphorylation on sites T568 and S572 upon infection with all variants. AAK1 regulates clathrin-mediated endocytosis by phosphorylating the AP2M1/mu2 subunit of the adaptor protein complex 2 (AP-2) which ensures high-affinity binding of AP-2 to cargo membrane proteins during the initial stages of endocytosis^46,47^. By regulating clathrin-mediated endocytosis, AAK1 plays a role in the entry of e.g., hepatitis C virus as well as the life cycle of other viruses such as Ebola and Dengue viruses^48^.

### Phosphorylation sites in SARS-CoV-2 proteins reveal potential functional impacts

When analyzing the phosphorylation sites in SARS-CoV-2 proteins, we noticed that N, ORF9b, and M proteins were the only viral proteins that were detected in our phosphoenriched MS results (Fig. 4b). The detected phosphosites, present in all the VOCs used in this study, validate many of the possibly phosphoregulated sites predicted based on protein sequence^32,49^. To predict the potential role of these phosphorylation events, we mapped the sites onto the respective protein 3D structures.

Since N protein lacks a comprehensive whole protein 3D structure, we utilized the N protein domains (PDB: 6WKP, 7PKU, and 6WZO) to predict the effect of phosphosites on the N protein structure and function. The majority of phosphosites were located in the N-terminal part of the N protein. In the near proximity of the RNA-binding region, we identified the phosphorylation sites T76 and S79, coinciding with the two mutations of interest, D63G and P80R, described below. Furthermore, we identified phosphorylation site Y268 in the putative NLRP3 binding region and in the proximity of the nuclear localization signal region. The NLRP3 is a protein involved in innate immune responses and phosphorylation in this region may modulate interactions with NLRP3, potentially affecting the host’s immune response to the virus infection. The observation of phosphorylation sites in the proximity to the nuclear localization signal region suggests a potential role in regulating the subcellular localization of SARS-CoV-2 N protein (Supplementary Fig. S1).^50–52^

We used AlphaFold 3 to predict the structures of human NLRP3, SARS-CoV-2 N protein, and the N protein dimerization domain (260–340 aa), which is suggested to bind to NLRP3^53^. While AlphaFold 3 could not accurately predict the full SARS-CoV-2 N protein structure (pTM = 0.39), it identified two domain-like structures, one of which likely contains the NLRP3 binding site, resembling the 6WZO structure. The N protein dimerization domain matched the 6WZO structure, validating the prediction. The NLRP3 structure prediction was more consistent (pTM = 0.64) and featured a ‘swan/dragon-like’ conformation with a half-barrel binding domain.

Attempts to predict NLRP3–N protein binding were inconclusive (pTM < 0.5) due to the uncertainty of the N protein structure. However, predictions using the N protein dimerization domain (with and without the Y268 phosphosite) showed some consistency (pTM = 0.5), with ~50% of the peptide binding to the NLRP3 half-barrel domain in a manner that positioned Y268 toward the barrel. This aligns with Pan et al.^53^ findings, suggesting that Y268 may play a role in binding.

We further validated the NLRP3–N protein interaction experimentally through co-immunoprecipitation (co-IP) using wild-type (WT) N protein and two phosphosite mutants (Y268D and Y268F). Dot blot analysis revealed that the N Y268F mutant showed stronger binding to NLRP3 compared to Y268D (*P* = 0.000006) and WT (*P* = 0.000007) N proteins. The substitution of tyrosine with phenylalanine (Y268F) eliminates the phosphorylation site, suggesting that the loss of phosphorylation at this position may enhance the interaction between the N protein and NLRP3. In contrast, the Y268D mutation, which mimics a phosphorylated state, showed weaker binding to NLRP3, indicating that phosphorylation of Y268 may hinder or reduce the interaction. The WT protein, which can be phosphorylated at Y268, also exhibited lower binding affinity compared to Y268F, supporting the idea that phosphorylation at this site modulates the interaction. These results suggest that phosphorylation of Y268 negatively regulates the N protein’s binding to NLRP3, likely by introducing electrostatic repulsion or altering the protein’s conformation, while the unphosphorylated form enhances this interaction through hydrophobic or aromatic interactions.

ORF9b has been identified as one of the key players in SARS-CoV-2 viral evolution and immune escape^2,54^. Here, we detected two specific phosphorylation sites, S50 and T72, within the ORF9b protein. Based on the molecular structure (PDB: 6Z4U), we observed that both phosphorylation sites were located approximately in the beginning of the β-sheet, closer to the protein core. This positioning suggests that these phosphorylation sites may play a role in ORF9b structural activation and morphological changes.

As for virus M protein, we observed a trend indicating that the 3′ intravirion end is a phosphorylation-rich region. This observation is expected, considering that M proteins often play a crucial role in intracellular signaling and cellular recognition processes.

### Overview of gene expression across SARS-CoV-2 variants

To examine the viral infection progression at the transcriptional level, we conducted gene expression analysis using NanoString technology, which enables direct quantification of RNA molecules without the need for reverse transcription or amplification. From our NanoString analysis (Supplementary Table S9 and Fig. S2), we quantified the expression of 456 genes, which were categorized into immune response/inflammation (174 genes), signal transduction (76 genes), transcription factors (68 genes), cell cycle/proliferation (38 genes), and apoptosis/cell death (18 genes). Each SARS-CoV-2 variant exhibited a distinct gene expression pattern. Beta variant samples demonstrated moderate to high expression of apoptosis and immune response genes, suggesting an active yet balanced immune response. In contrast, Alpha variant samples showed elevated expression of inflammatory and apoptosis-related genes, indicating a more aggressive immune response associated with this variant. Delta variant samples exhibited particularly high expression levels of both immune response and apoptosis genes, reflecting a strong and aggressive immune response that may contribute to the severe clinical outcomes often observed with COVID-19 infections caused by this variant^55^. BA.1 samples displayed varied gene expression profiles, with some samples showing high levels of cell adhesion and migration genes. Interestingly, BA.1 also showed moderate expression of inflammatory genes, indicating a less aggressive inflammatory response compared to Delta variant. BA.5 samples were characterized by high expression of immune-related and apoptosis genes, reflecting an immune response.

During the progression of infection, gene expression of the panel genes was initially low, corresponding to the early stages of the host response. By 8 hpi, there was a noticeable increase in immune response gene expression across all variants, marking the onset of the host immune response. By 16 hpi, gene expression profiles revealed a stronger immune response with significant upregulation of inflammatory and antigen presentation genes, especially in Delta and Alpha variants. By 36 hpi, gene expression peaked for many apoptosis and inflammatory genes, particularly in Delta and BA.5 samples, indicating that the immune response had reached its peak activity. The increase in transcription levels at 16 hpi supports the selection of 36 hpi as a later time point for analysis.

### Molecular cloning of the SARS-CoV-2 variant-specific viral ORF variants and mapping their host–cell interactions

**Fig. 5:**
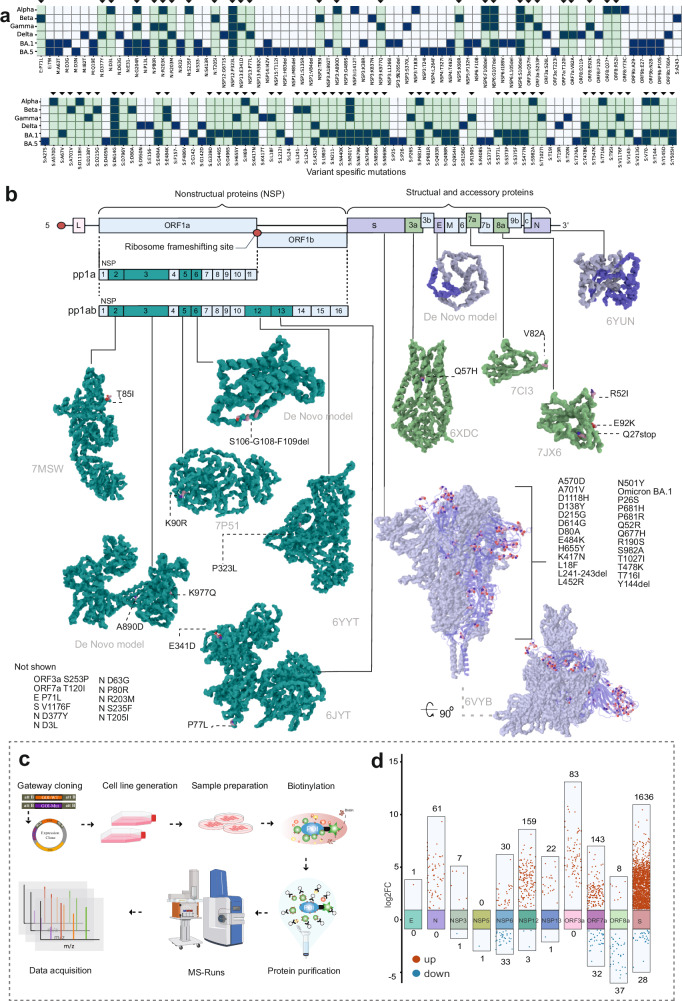
Protein–protein interaction changes detected for the 50 mutations in the SARS-CoV-2 variants. **a** An overview of all mutations detected in SARS-CoV-2 variant proteins (dark blue). Mutations selected for this study are highlighted in green and small arrow at the top of the columns. **b** Genomic architecture of SARS-CoV-2, which includes 14 ORFs encoding structural proteins (S, E, M, and N), non-structural proteins (pp1a and pp1ab), and nine accessory proteins. Structures of 12 selected viral proteins with their corresponding PDB IDs (RCSB PDB database) are shown, with variant-specific mutations highlighted in light pink. Structural proteins are shown in slate blue, and other ORFs in limon. NSPs cleaved from pp1a and pp1ab are depicted in teal. **c** Schematic illustration of the BioID pipeline. The MAC-tagged viral ORF generates an inducible ORF-expressing cell line, and interacting proteins are purified using single-step affinity purification, followed by MS analysis. **d** Multi-group difference scatter plot derived from PPI data, comparing mutations in each viral ORF to the WT viral ORF at the MS1 level. Significant changes in protein levels were identified, and these proteins were cross-referenced with a high-confidence list of interacting proteins from spectral count (MS2) data. The scatter plot displays log_2_FC values on the *y*-axis against comparison groups on the *x*-axis with the number above (upregulated) or below (downregulated) each bar indicating the number of significant interactions with the viral protein.

To understand the effect of mutations in VOCs, we assessed the mutation-induced changes in virus–host protein interactions. We analyzed the sequences of the five SARS-CoV-2 variants (GISAID.Org 2024) and identified and selected 50 distinct mutations of interest affecting 12 viral proteins (NSP2, NSP3, NSP5, NSP6, NSP12, NSP13, S, ORF3a, E, ORF7a, ORF8a, and N) (Fig. 5a; Supplementary Table S1). We mapped the selected VOC mutations onto the structures of SARS-CoV-2 proteins (structures obtained from the RCSB Protein Data Bank (RCSB PDB), imaged by pyMol) (Fig. 5b).

To further study effects of mutations on viral ORFs, we conducted proximity-labeling (BioID) experiments coupled with liquid chromatography-mass spectrometry (LC-MS) analysis to investigate possible alterations in protein–protein interactions (PPIs). The proximity labeling approach is advantageous due to its ability to capture also weak or transient interactions spatiotemporally and to allow the interactome analysis in the native cellular context^14,56,57^. We generated SARS-CoV-2 ORF variant-expressing isogenic tetracycline-inducible HEK293 cell lines and subjected them to our BioID pipeline (Fig. 5c).

Our BioID analysis yielded a comprehensive dataset of totally 5654 high-confidence interactions (HCIs) and 1151 high-confidence interacting proteins (HCIPs) with the 12 Wuhan-Hu-1 (WT) viral ORFs and the 50 mutated SARS-CoV-2 ORFs (Supplementary Table S6). A comparative analysis was carried out to assess the protein interactions of mutated viral ORFs in contrast to the WT ORFs (Fig. 5d). Predominantly, the increased number of interactions was observed to concentrate around the S protein, which is consistent with the extensive variety of variants in this protein. The data also reveal a significant increase in interactions with the mutated N protein, NSP12, NSP13, ORF3a, and ORF7a. Interestingly, with ORF8a we detected decreased number of interactions with the mutants. This decreased number of interactions with the mutated ORF8a, is likely attributable to the Q27stop mutation, which effectively leads to the loss of ORF8a functionality. This also correlates with our earlier findings (Fig. 1d), where we mostly observed a very low levels of ORF8a in Alpha and Omicron BA.5 carrying the Q27stop mutation.

**Fig. 6:**
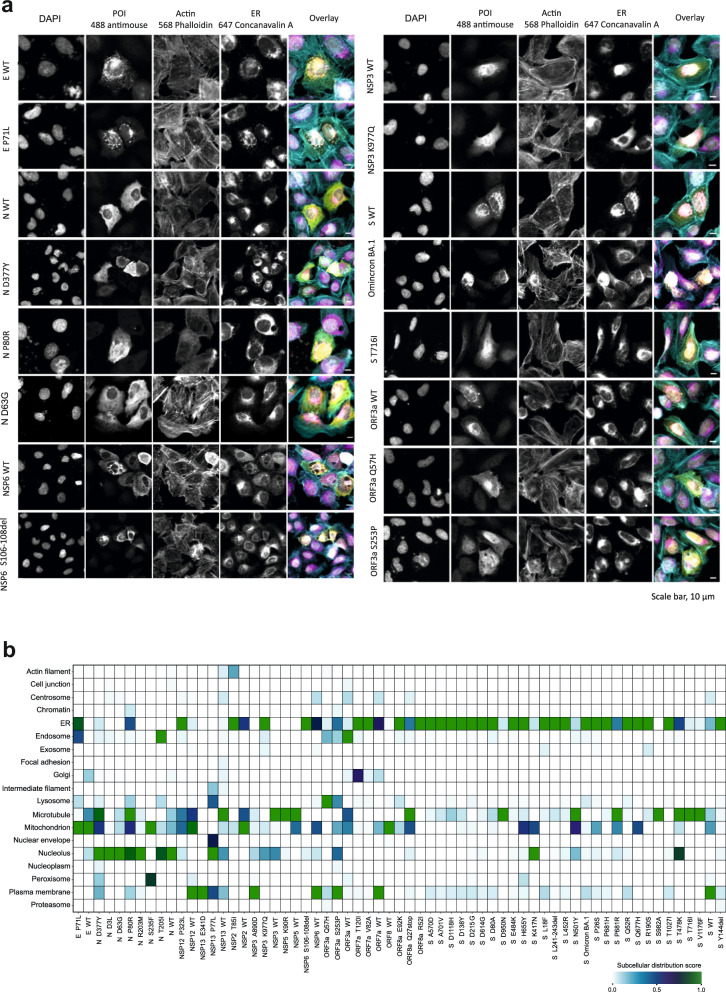
Molecular microscopy and immunofluorescence analysis of the SARS-CoV-2 mutation localization and induced cellular phenotypes. **a** Immunofluorescence microscopy images of the SARS-CoV-2 proteins expressed from the MAC-tagged viral ORFs, transfected into U2-OS cells, and detected by immunofluorescence. Scale bars, 10 µm. **b** MS-microscopy analysis heatmap of the mutations from VOCs, illustrating the estimated localization of the bait protein based on proximity-dependent biotin identification coupled with MS interaction results.

Additionally, to analyze the expression, subcellular localization and the potential morphological changes induced by the viral proteins, we transfected the previously generated MAC-tagged versions of the SARS-CoV-2 ORFs into U2-OS cells (Fig. 6a, b). To detect the ORF expression, we employed the HA-epitope within the MAC-tag. To evaluate the general cell morphology and cytoskeleton integrity, we utilized Phalloidin staining, and to visualize the ER, we used Concanavalin A staining. The expression of ~80% (49 of 62 constructs) of the SARS-CoV-2 ORF constructs was verified by the immunofluorescence analysis, with non-productive or toxic expression of some S mutants (Fig. 6a; Supplementary Table S5, Fig. S3).

Wuhan-Hu-1 (WT) E protein and P71L mutated E protein were both well expressed in the U2-OS cells. The N protein mutants, D63G, P80R and D377Y were also expressed comparably to the WT N protein. The N protein P80R and D377Y mutations, unique to the Delta variant, resulted in a localization shift in nucleus/cytoplasm equilibrium (towards the nucleus). We noted a distinct difference in the NSP6 S106–108del mutant compared to WT NSP6. Specifically, the cells expressing S106–108del mutant exhibited smaller budding vesicle-like structures, contrasting with the larger vesicle-like structures observed in the cells expressing WT NSP6, confirming the observation described also previously^58^.

As shown in our previous study^56^, NSP3 induces nuclear actin localization, now both in cells expressing WT and K977Q mutated NSP3.

The expressed S proteins were mostly localized to the ER, except the T716I mutated S protein, which showed an enhanced cytoplasmic localization. Our previous study described the localization of ORF3a around the ER-Golgi region^56^, and now, with the use of specific ER staining, we can confirm that ORF3a is also localized in the ER. Furthermore, our results suggest that the ORF3a proteins mutated at Q52H and S253P have different localization patterns, being more cytoplasmic compared to the Wuhan-Hu-1 ORF3a (Fig. 6a).

Using the MS-microscopy approach, which utilizes the BioID HCI data to assign tested proteins to a subcellular location, we obtained high localization correlation with our immunofluorescence microscopy analysis (Fig. 6b). Combining and comparing the BioID and microscopy data provides a robust validation of our findings.

### Overlapping pathways are affected by mutations in SARS-CoV-2 VOCs in the interactome, proteome and phosphoproteome datasets

**Fig. 7:**
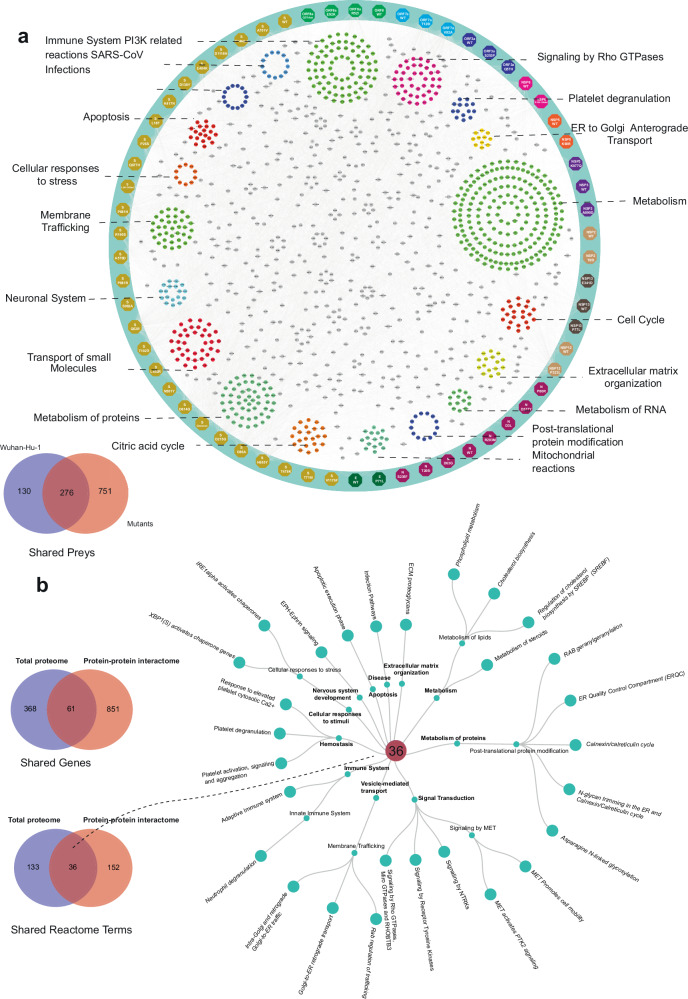
SARS-CoV-2–host physical and functional interaction knowledge graph. **a** SARS-CoV-2 high-confidence interactome map with the 12 WT and 50 mutated SARS-CoV-2 proteins. The enriched Reactome term clusters display interactions with preys with the corresponding terms. The Venn diagram showing the prey overlap detected by WT and mutated ORFs. **b** The circular dendrogram of 36 Reactome pathways depicts the overlap of Reactome terms between the two datasets. The Venn diagrams illustrate shared proteins and Reactome terms between the global proteome dataset and the BioID dataset.

Delving more in depth in BioID results, we analyzed the whole SARS-CoV-2 interactome with the host cell proteins. We analyzed the interactions formed by the 12 SARS-CoV-2 ORFs and the corresponding 50 mutants with the host proteome. In total, the 62 ORF constructs yielded 1151 HCIPs of which 11% were WT ORF-specific, 24% were shared by the WT ORFs and mutated ORFs, and 65% were mutated ORF-specific. To generate an almost complete SARS-CoV-2–host interactome, we combined all of the SARS-CoV-2 ORF HCIs with HCIPs to an interactome knowledge graph depicting the interactor specific Reactome pathways (Fig. 7a; Supplementary Table S6). Similar pathways, connected with the SARS-CoV-2, were detected with the ORFs as in the global proteome and phosphoproteome analyses of the SARS-CoV-2 VOC-infected cells. The most commonly detected pathways with SARS-CoV-2 ORFs include ‘Metabolism’ (192 proteins), ‘Immune System’ (79), ‘Metabolism of proteins’ (52), ‘Signaling by Rho GTPases’ (41), ‘Transport of small molecules’ (38), and ‘Membrane Trafficking’ (36). Comparison of the BioID Reactome results with the total proteome Reactome findings (Fig. 2), resulted in identification of 61 unique proteins shared between BioID data (851 unique proteins) and total proteome dataset (368 unique proteins) (Fig. 7b; Supplementary Table S6). In Reactome terms this translates to 36 unique Reactome pathways, such as ‘ER Quality Control Compartment (ERQC)’, ‘N-glycan trimming in the ER and Calnexin/Calreticulin cycle’, ‘Signaling by Rho GTPases’, and ‘Neutrophil degranulation’.

Our comprehensive data on the total proteome, phosphorylation, and PPIs in both SARS-CoV-2 variant-infected cells and SARS-CoV-2 protein-expressing cells highlights key cellular pathways involved in SARS-CoV-2 infection and reveals a multitude of specific, critical interactions that may inform new therapeutic strategies.

### Druggability assessment for the key proteins in the SARS-CoV-2 ‘hijackome’ reveals novel druggable target candidates

To explore the potential to develop new therapeutic strategies based on our findings, we compiled a list of 1474 proteins of interest. This list includes all target candidates that showed significant changes in protein expression and phosphorylation in infected cells, as well as prey proteins identified with high confidence in the PPIs dataset (Supplementary Table S9). We analyzed their potential druggability by a combined structure-based druggability prediction and knowledge mining approach. In the structure-based analysis, we screened 12,153 protein structures for the presence of druggable pockets: 6531 X-ray crystal structures, 4172 electron microscopy (EM) structures, and 1450 AlphaFold models. Fifteen proteins were excluded from the analysis, since no structural data were available. Initially, we focused on conventional druggable sites, since, as also discussed in our previous study, ligand binding can induce conformational changes resulting in the possible perturbation of PPIs^56,59^. 1126 of the targets were predicted to be likely druggable by drug-like small molecules targeting well-enclosed pockets (Supplementary Table S9). In contrast, when attempting to directly disrupt PPIs, targetable sites are typically shallower and more hydrophobic, which we accounted for with a modified pocket screening protocol^60^. We found 680 targets with potential PPI sites, which were predicted to be druggable or likely druggable by small molecules. Sites with volumes of 160–800 Å^3^ were considered druggable, but we also detected several potential PPI sites with larger volumes. Those are unlikely to be directly druggable by small drug-like molecules but might still be amenable to targeting approaches with other types of interactors or could be addressed by targeting subsites. 61 targets further showed potentially druggable cryptic sites: Their initial volume was below 160 Å^3^, but it was possible to induce the opening of the sites with a ligand molecule, using a modeling protocol accounting for limited protein flexibility^60^ (Supplementary Table S9).

We additionally analyzed bound ligands in all experimental structures for their properties and found that 273 proteins had been previously described to bind one or more ligands satisfying Lipinski’s Rule-of-Five (RoF) as a metric for drug-likeness of orally bioavailable compounds^61^. Inhibitors of PPIs (iPPIs), on the other hand, often violate RoF, since they have higher molecular weight and are more hydrophobic^62^. A further 114 proteins of interest contained ligands with a property profile more similar to iPPIs (Supplementary Table S9). Finally, based on the literature analysis and the Therapeutic Target Database (TTD)^63^, 69 of the proteins can be considered established targets with examples for successful drug design or patenting, 91 are currently being explored in clinical research and another 129 have at least been previously studied to some degree (Supplementary Table S9). The full druggability analysis workflow is schematically depicted in Supplementary Fig. S4.

Focusing on the 60 proteins which showed major changes in protein expression, phosphorylation and PPIs, we predicted 46 to possess likely druggable deep binding sites, and 14 to have druggable PPI sites. Of the predicted proteins, 17 of them had high to medium druggability confidence, were mostly researched and established targets with only three exceptions, and accounted for 16 and 5 druggable deep and PPI sites, respectively. However, with few exceptions, such as histone deacetylase 6 and hexokinase-2^64–66^, even those particularly likely druggable target candidates have so far not been studied in depth in context of their potential role in, and impact on, infections with SARS-CoV-2 or other coronaviruses. Additionally, even when just considering the most robust target candidates highlighted by our experimental studies, half of the to date unexplored proteins were predicted to have potential druggable deep binding sites, and almost one third potential iPPI-targetable sites. Drawn from the druggable ‘hijackome’ of SARS-CoV-2, those, as well as several candidates flagged mainly by protein expression and proteomics or PPI studies, may represent particularly promising starting points for the development of novel anti-coronaviral strategies.

## Discussion

The current investigation into SARS-CoV-2 variants’ interactions with host cells underscores the complexity of viral infection dynamics and host–pathogen interplay. By employing a comprehensive multi-omics approach, we systematically characterized the temporal and variant-specific alterations in host cell proteins and cellular processes upon infection with SARS-CoV-2 VOCs, namely Alpha (B.1.1.7), Beta (B.1.351), Delta (B.1.617.2), and Omicron BA.1 and BA.5. The variants in our analysis have allowed us to observe similarities and certain differences in infection spatiotemporal dynamics. These observations may partially explain variant-specific differences in pathogenesis and can be considered in developing strategies to combat SARS-CoV-2. This study’s breadth, spanning quantitative proteomics, phosphoproteomics, and interaction proteomics, offers an extremely broad view into the molecular mechanisms underpinning SARS-CoV-2 variant pathogenicity and host defense systems. By employing quantitative proteomics and phosphoproteomics, we gained valuable insights into how SARS-CoV-2 VOCs impact cellular signaling networks throughout the entire course of infection, from 1 hpi to 36 hpi. The inclusion of the 36 hpi time point seems especially important for understanding the progression of infection in its later stages. It reveals the changes of transcription factors’ activities, providing insight into how the virus modulates host transcriptional responses and signaling networks during prolonged infection times. This likely correlates with immune responses or viral evasion strategies, as well as cellular stress or the onset of cell death. By integrating data on phosphorylation changes in cellular signaling molecules with variant-specific mutations in viral ORFs and their impact on protein expression, as detected by proximity labeling, we generated a comprehensive functional and mechanistic view of SARS-CoV-2 infection. This wealth of information enhances our understanding not only of SARS-CoV-2 infection but also of variant-induced changes. The knowledge base serves as a foundation for further analyses and potential drug discovery approaches.

However, we acknowledge several limitations in our analysis, including the analysis of a limited number of variants, which may not encompass the full genetic diversity of the most recent circulating SARS-CoV-2 strains after BA.5. Moreover, the investigation of each mutation individually may overlook potential co-effects of mutations, as well as co-factors and interactions among viral proteins during infection. Additionally, the use of only one cell line does not fully represent the large and diverse set of cell types and tissues targeted by SARS-CoV-2. Furthermore, the predominantly in vitro experiments may not completely replicate the complex conditions of viral infection in vivo in an organism, and the direct applicability of the findings to other coronavirus types and new variants may be limited.

### The role of Rho GTPase signaling in SARS-CoV-2 variant-infected cells

The consistent upregulation of Rho GTPase signaling components across SARS-CoV-2 variants suggests a conserved viral strategy to remodel the host cell cytoskeleton, promoting viral entry, replication, and release. In contrast, the variant-specific phosphorylation patterns observed in kinases and phosphosites point to diverse regulatory mechanisms employed by different VOCs to hijack host cell signaling, highlighting the virus’s evolutionary adaptations. Our research identified specific mutations and proteins that enhance Rho GTPase cycle regulation and association (Fig. 7a). Reactome enrichment from BioID analyses emphasized the significance of viral protein interactions with Rho GTPase signaling. Key mutations — including S-protein mutations (D138Y, D215G, D80A, E484K, L241–243del, L452R, P681H, R190S, Y144del), NSP12 (P323L), ORF3a (Q57H), ORF7a (T120I, V82A), and NSP6 (S106–108del) — were identified in both total proteome and comparison analysis results relative to Wuhan-Hu-1 (WT). The Rho GTPase pathway, regulated by Rho GTPases, is crucial in numerous cellular processes^67^. Activation or alteration in Rho GTPase expression in cells expressing mutations in S, NSP6 (Alpha, Beta, Gamma, Omicron BA.1/5), and ORF7a (Delta) may trigger distinct signaling pathways and cellular responses. Rho GTPases play multiple roles in SARS-CoV-2 infection^68^, including enhancing viral entry and ACE2 receptor regulation, the primary receptor for viral entry^68,69^. Activation of RhoA and Rac1, for example, modulates ACE2 expression, potentially increasing infection rates^69,70^. SARS-CoV-2 internalization requires actin cytoskeleton remodeling, to which Rho GTPases contribute^70^. Additionally, Rho GTPases regulate intracellular trafficking, essential for viral component trafficking and replication compartment formation^71^. They also modulate immune responses during coronavirus infection by regulating proinflammatory cytokine production and immune cell migration, bolstering the host response against the virus^72^. Notably, Rho GTPase signaling is activated in various viral infections, including respiratory syncytial virus and Ebola virus infections^73–75^.

### Exploitation of ERAD pathway for viral replication and host cell hijacking

Additionally, the GO enrichment analysis for biological processes demonstrated a strong association between certain SARS-CoV-2 VOC mutations, notably NSP12 (P323L), ORF8a (E92K), and S (A570D, A701V, D80A, L18F, P681H, P681R, R190S, T478K), with biological processes related to the ERAD and ER-related pathways (Fig. 7a; Supplementary Tables S7, S8). Our global quantitative proteome data further confirmed an upregulation of the ERAD pathway linked to these mutations, consistent with findings from our previous study^56^.

In the ERAD pathway, the virus is expected to enter host cells and replicate in the ER-Golgi intermediate compartment (ERGIC) and ER. During the viral replication cycle, the SARS-CoV-2 produces a large number of viral proteins, some of which may be misfolded or aberrantly processed^76,77^. The ERAD pathway recognizes these misfolded viral proteins and targets them for degradation to prevent their accumulation, which can disrupt normal cellular processes and induce cellular stress responses^78^. An increase in the expression of several components of the ERAD pathway has been implicated in the response to SARS infection. For example, studies have shown that the SARS-CoV-2 triggers the unfolded protein response in infected cells, which is a cellular stress response that upregulates ERAD components to cope with the increased misfolded protein load and restore ER homeostasis^30,79^.

### Manipulation of host mRNA dynamics and viral replication machinery

In addition, mechanisms related to mRNA and RNA processing play a crucial role in the SARS-CoV-2 life cycle. Notably, SARS-CoV-2 has demonstrated its ability to interfere with host cell mRNA splicing and mRNA stability. This viral strategy effectively prioritizes the translation of viral mRNAs over host cell mRNAs, enabling the virus to take over the host cell protein synthesis machinery for its own replication^80^. Furthermore, SARS-CoV-2 exhibits the capacity to modulate host cell RNA processing and mRNA stability to evade the host immune responses. For instance, by perturbing mRNA splicing and stability, the virus can reduce the production of interferons (IFNs), which are essential components of the host’s antiviral defense^81^. We also detected several connections of SARS-CoV-2 infection with host cell mRNA regulation. In NSP13 viral mutant expressing cells, a decrease in innate immune response to viral infection was observed, indicating a potentially important role in immune evasion^82^. NSP13 is primarily involved in unwinding and separating double-stranded RNA during viral replication^82^. It is considered an attractive target for antiviral drug development as it plays a critical role in viral replication^83^. NSP12, on the other hand, catalyzes the synthesis of newly synthesized RNA molecules using the viral RNA genome as a template^84^. NSP12 is also an essential enzyme for viral replication and a target for antiviral drug development^85,86^. Drugs like remdesivir that target NSP12 have shown efficacy in treating COVID-19 patients^87^. In GO term interactome analysis, the NSP12 P323L mutation, present in all variants, showed more connections with the citric cycle regulation and innate immune system compared to the WT NSP12 protein. The NSP12 P323L mutant also exhibited interaction with proteins regulating innate immune responses and negative regulation in apoptotic processes. Such interactions were also seen for NSP13 ORF variants.

Previous studies have shown that SARS-CoV-2 ORF6, ORF8, and N proteins are potent IFN antagonists^88–90^. Additionally, NSP13, ORF3b, ORF9b and ORF7a have also been found to exhibit ubiquitination and type I IFN antagonism^88,90,91^. One study linked ORF7a to the inhibition of type I IFN gene expression^92^. Another study found that the K119A mutation in ORF7a reduces its antagonism of IFN signaling, suggesting that ubiquitination at K119A is crucial for downregulating the host antiviral response^91^. Similarly, nearby mutations, such as T120I, may also impact IFN response. In our Reactome enrichment analysis, pathways regulating RNA and innate immune responses were significantly enriched among interactors of ORF7a mutants (V82A and T120I). Mutations in ORF7a, especially in the Delta variant, may therefore contribute to the increased dominance of these variants over earlier strains. These findings imply that specific mutations in SARS-CoV-2 proteins, particularly those that downregulate IFN response, could drive the emergence and success of new viral variants.

The N protein of SARS-CoV-2 primarily functions in packaging the viral genome and has an RNA binding ability^89,93^. The N protein has been suggested to act as a viral suppressor of RNAi in other coronaviruses, inhibiting IFN production. The N protein interacts with the helicase domain of RIG-I leading to reduced IFN-β gene expression due to reduced recognition of viral RNA by RIG-I^89,93^. Mutations in the N protein show upregulation of cellular proteins involved in translation and RNA binding, as well as immune evasion, suggesting an increased ability of viral N protein-expressing cells for immune evasion^94^. Due to its key role in RNA binding and viral replication, the N protein is a potential target for drug development^95^. Considering the structural and phosphosite information, we can predict N protein mutations D63G and P80R to have importance in immune evasion. These mutations are commonly seen in Gamma and Delta variants.

### Building a more complete view on the SARS-CoV-2 host cell ‘hijackome’

Several proteomics analyses have been performed on SARS-CoV-2 virus-infected cells. In an earlier study by Klann et al.^13^, the authors identified an enhanced expression of GFR signaling components, an observation that was also verified in our study. However, being an early study, it primarily focused on the Wuhan SARS-CoV-2 strain and was limited to a single time point of 24 h. Another study by Stukalov et al.^14^ expanded the investigation further by having multiple time points up to 36 h, observing later stages of viral infection and reaffirming interactions related to the GFR signaling pathway. A study conducted by Thorne et al.^15^ focused specifically on the Alpha variant, revealing its increased ability to suppress innate immune responses compared to an ancestral virus variant. Utilizing proteomics and RNA sequencing, they identified increased levels of specific viral proteins known to antagonize the innate immune response, suggesting a mechanism for the Alpha variant’s increased transmission.

A study by Bouhaddou et al.^96^ aimed to understand different SARS-CoV-2 variants’ effects on viral replication and cellular responses using mRNA sequencing, AP-MS, and sgRNA phosphoproteomics. They found three convergent molecular strategies across variants, altered viral gene expression, modulation of viral protein phosphorylation, and protein-coding mutations affecting virus–host interactions. Despite seemingly similar to the study by Bouhaddou et al.^96^, our research offers unique insights; while they used AP-MS, we employed proximity labeling that, based on our previous study by Liu et al.^56^, was found to be effective in capturing transient interactions, and more suitable for studying host–pathogen signaling than AP-MS. Our study mainly focused on variants delivered from Finland (Alpha, Beta, Delta, Omicron BA.1, and Omicron BA.5), while Bouhaddou et al.^96^ included a broader range of globally circulating variants. Our study also includes the more recent BA.5 variant and additional time points up to 36 hpi. The need for later time point analyses were suggested by Stukalov et al.^97^, which highlighted the importance of later than 16 h stages post infection for protein level analyses. Our time points of 1 hpi, 8 hpi, 16 hpi, and 36 hpi provide a more dynamic understanding of the infection process from start to end, though our focus was to show the less studied 36 hpi virus dynamics.

Liu et al.^97^ conducted similar phosphopeptide enrichment in rhesus monkeys, inspecting SARS-CoV-2. We observed overlap between Bouhaddou et al.^96^, Liu et al.^97^ and our study in global proteomics data, with ~50% overlap among the three studies, each having unique identifications. However, phosphopeptide data were less comparable between studies, and additionally, Liu et al.^97^ did not have any variant data. Nonetheless, the combination of datasets from Calu-3 and VeroE6 cell models, along with rhesus monkey data, provides a more comprehensive understanding of SARS-CoV-2 infection and host–pathogen interactions. Additional studies are still needed to delve deeper into host responses, especially with the emergence of new variants.

Additionally, in this study, we employed an extensive analysis of the potential druggability across the SARS-CoV-2 host cell ‘hijackome’. Our analyses highlighted a considerable number of key proteins as established druggable or actively studied targets, but also pointed to over 800 target candidates with predicted druggable sites amenable to modulation by classical drug-like molecules and over 500 with small-molecule druggable PPI sites, all of which appear to be presently largely unexplored. Our analysis indicates that many of the most promising target candidates, identified for their significant changes in protein expression, phosphorylation, and PPIs, have high druggability potential but have not been extensively studied in the context of SARS-CoV-2. Approximately half of these key proteins are predicted to be druggable yet remain unexamined, presenting numerous opportunities for novel ‘hijackome’-targeting strategies that merit further investigation.

## Conclusion

In summary, our extensive proteomic analysis of SARS-CoV-2 variants using quantitative proteomics, phosphoproteomics, and proximity-labeling interactomics revealed variant-specific differences in host protein interactions, expression, and activation of cellular signaling networks. Examining Alpha, Beta, Delta, BA.1, and BA.5 variant infections in in vitro cultured cells, we observed differences in replication kinetics among variants, as well as distinct capacities to modulate host protein expression and activation. Key components of the Rho GTPase cycle, RNA splicing, and ERAD-related processes showed enhanced expression and activation, along with variant-specific alterations. Our findings shed light on the dynamic regulation of cellular pathways during SARS-CoV-2 infection and identify potential targets for new therapeutic interventions. This research significantly advances our understanding of SARS-CoV-2’s interactions with host cell proteins at the proteomic level, providing a foundation for further mechanistic and molecular studies of this important virus.

## Materials and methods

### Cloning

Entry clones containing genes of interest were obtained from the ORFeome collection (ORFeome and MGC Libraries; Genome Biology Unit supported by HiLIFE and the Faculty of Medicine, University of Helsinki, and Biocenter Finland). The gene of interest used for generating the stable cell lines was fused to MAC-tag-C (Addgene, Plasmid #108077) destination vector using Gateway cloning techniques. The plasmids used in this project are available from Addgene. The gene of interest used for protein interaction validation was fused to a modified pDEST40 vector (with a 3× V5 C-terminal tag) using Gateway cloning techniques. Point mutations of SARS-CoV-2 variants were created by performing site-directed mutagenesis to plasmids using Q5 high-Fidelity DNA polymerase (NEB, M0491L). Primers were obtained from Eurofins Genomics. Mutations of interest were designed based on GISAID database (GISAID.ORG) and WHO classification of variant of concern/interest including Alpha (B.1.1.7 and Q lineages), Beta (B.1.351 and descendent lineages), Gamma (P.1 and descendent lineages), Delta (B.1.617.2 and AY lineages), and Omicron (B.1.1.529, BA.1, lineages). Final selection of the mutation of interest (50 constructs) was based on the significant occurrence of mutation within variant strains.

### Cell culture

The Flp-In™ 293 T-REx cell line (Invitrogen, R78007), HEK293 cell line (ATCC® CRL-1573™), and U2-OS (ATCC®, HTB-96™) were maintained under the manufacturer-recommended conditions. Transfection and generation of stable cell lines were performed as previously described^57^. Briefly, Flp-In™ 293 T-REx cells were co-transfected with the MAC-tagged bait or GFP construct and pOG44 vector (V600520, Invitrogen) using FuGENE 6 transfection reagent (Promega, Wisconsin, USA). At 48 hpi, cells were selected for 3 weeks in medium containing hygromycin B (100 μg/mL; Invitrogen). The positive clones, containing stable isogenic MAC-tagged baits, were further expanded to 80% confluence in 10 × 150-mm cell culture plates (CELLSTAR, Greiner) in complete culture medium. Cells from five plates were used for one biological replicate, and to induce the expression of protein of interest, 1 μg/mL tetracycline (Sigma-Aldrich, T3383-25G) was added, together with 50 μM biotin (Thermo Fisher Scientific, 29129) for 24 h before harvesting.

### Virus preparation

SARS-CoV-2 variants were isolated from nasopharyngeal samples in VeroE6 or VeroE6-TMPRSS2-H10^98^ cells: FIN34-21 (Alpha variant; GenBank ON532062.1), FIN32-21 (B.1.351, Beta variant; OK448476.1 and EPI_ISL_3471851), FIN37-21 (B.1.617.2, Delta variant, OK626882.1 and EPI_ISL_2557176) and Omicron variants FIN56-21 (BA.1.1 variant, EPI_ISL_8586102) and FIN61-22 (BA.5 variant, OP435368 and EPI_ISL_13118918). Viruses were further amplified in VeroE6-TMPRSS2-H10 cells in DMEM supplemented with 2% fetal calf serum, 2 mM L-glutamine, and 2 mM penicillin-streptomycin. Virus stocks were tittered in VeroE6-TMPRSS2-H10 cells by median Tissue Culture Infectious Dose (TCID_50_) assay as described before^99,100^.

### Infection experiments

For infection, VeroE6-TMPRSS2-H10 cells were seeded on the previous day (3 × 10^6^ cells per 10-cm^2^ dish, 0.4 × 10^6^ cells per 6-well plate well, and 0.075 × 10^6^ cells per 24-well plate well) in DMEM supplemented with 10% fetal calf serum, 2 mM L-glutamine, and 2 mM penicillin-streptomycin. At the time of infection, the medium was changed to DMEM supplemented with 2% fetal calf serum, 2 mM L-glutamine, and 2 mM penicillin-streptomycin, and cells were kept in this medium for the rest of the time. Cells were infected with SARS-CoV-2 variants in half of the designated, final medium volume. Following infection, the cells were incubated in 37 °C and 5% CO_2_ for 1 h, after which the remaining half volume of the infection medium was added. Note that the inoculated virus was not removed. Depending on the experiment and the virus, the final titers were 20,000–70,000 TCID_50_/mL. In proteome and RNA analyses, the final titers were: 70,000 TCID_50_/mL Fin-34, 58,000 TCID_50_/mL Fin-37, and 25,000 TCID_50_/mL for Fin-32, Fin-56 and Fin-61. For immunofluorescent analyses, the final titers were 56,000, 46,400 and 20,000 TCID_50_/mL, respectively. Protein and RNA samples were collected and cells for immunofluorescence were fixed at time points of 1 hpi, 8 hpi, 16 hpi and 36 hpi. Samples from uninfected cells were collected as controls.

### Sample preparation from the infected samples

We plated 3 × 10^6^ cells per 10-cm^2^ dish (MOI of 0.1–0.2) on the previous day based on the cell count on the plating day. For protein samples, the cells were infected with 3–8 × 10^5^ TCID_50_ of variant per 10-cm^2^ plate. At the time of sample collection, the plates were kept on a cold plate, the medium was removed, cells were washed once with ice-cold TBS, 0.5 mL ice-cold freshly made 8 M urea solution (in 50 mM NH_4_HCO_3_ supplemented with Phosphatase Inhibitor Cocktail (Roche)) was added per 10-cm^2^ dish, and the cells were scraped off the plate. Cell lysates were kept on ice and stored at –80 °C until analysis.

### Infection rate estimation with immunofluorescence

For immunofluorescence, the cells were infected with 0.1–0.28 × 10^5^ TCID_50_ of variant per 24-well plate well. The cells were fixed with 4% formaldehyde for 1 h, the plates were washed with phosphate-buffered saline (PBS), and stored in PBS at 4 °C until analysis. The fixed cells were blocked and permeabilizated with 0.5% bovine serum albumin (BSA, Sigma-Aldrich) and 0.1% Triton X-100 (Sigma-Aldrich) in PBS (Gibco) for 30 min at room temperature. Subsequently, primary antibodies of in-house SARS-CoV-2 rabbit anti-N (gift from Sari Maljanen and Ilkka Julkunen) and rabbit anti-S1 antibodies^101^, diluted in 3% BSA in PBS, were added for 1 h at room temperature. The cells were washed thrice for 10 min with PBS. Secondary antibodies (Goat α-rabbit IgG (H + L) Alexa Fluor Plus 488, Thermo Fisher Scientific) and 4′,6-diamidino-2-phenylindole (DAPI, Life Technologies), both diluted in 3% BSA in PBS, were applied to the cells for 1 h at room temperature in dark. The cells were washed thrice for 10 min in 3% BSA in PBS. Subsequent imaging was performed utilizing the EVOS FL Auto imaging system (Life Technologies). The images were processed in Fiji, a common, open-source distribution of ImageJ^102^. To estimate the infection percentage, the nuclei were segmented using the StarDist2D plugin in Fiji^103^. A binary classifier was trained using the Trainable Weka Segmentation plugin^104^ in Fiji to identify which cells were infected and which were not. Roughly 20% of the images taken in the first repetition were used for training the algorithm and the remaining 80% of the images from the same repetition were used for validation. The resulting algorithm was then used on subsequent repetitions and image sets.

### NanoString platform analysis for immune response profiling

For the RNA samples, cells were infected with 0.5–1.4 × 10^5^ TCID_50_ of the variant per well in a 6-well plate. At the time of sample collection, the plates were placed on a cold surface, the medium was removed, and the cells were washed once with ice-cold PBS. Subsequently, 0.3 mL of RA1 buffer (Macherey-Nagel) containing 20 mM DTT was added to each well, and the cell lysate was gently resuspended by pipetting. The cell lysates were then kept on ice and stored at –80 °C until further analysis. RNA was extracted using the RNeasy® Mini Kit (Qiagen, 74004, Hilden, Germany). Approximately 100 ng of RNA was then analyzed using the NanoString nCounter gene expression platform (NanoString Technologies, Seattle, USA). The RNA was hybridized with the NanoString nCounter® Human Auto-immune Profiling panel (770 transcripts) according to the manufacturer’s instructions. Hybridization was performed on the nCounter Prep Station (NanoString Technologies), and the pre-loaded cartridges were scanned three times using the NanoString Digital Analyzer. The resulting gene expression data were analyzed with nSolver™ 4.0 analysis software (NanoString Technologies). Positive controls were used to account for potential sample variations, and data normalization was performed using housekeeping genes included in the panel, along with negative control subtraction.

### Quantitative proteomics and phosphoproteomics sample preparation

Viral-infected cell samples were lysed in lysis buffer 8 M Urea, 50 mM NH_4_HCO_3_ supplemented with 1× phosphatase and protease inhibitors cocktail (Sigma-Aldrich, P2745 and P8340) on ice. The lysate was cleared by centrifugation at 16,000× *g* for 10 min at 4 °C. The protein concentration was determined using a BCA protein assay kit (Thermo Fisher Scientific). Equal amounts of protein (250 µg) were obtained for all samples. Proteins were reduced with Tris(2-carboxyethyl) phosphine (TCEP; Sigma Aldrich), and alkylated with iodoacetamide. Samples were diluted by fourfold (to < 2 M urea) with 50 mM ammonium bicarbonate (AMBIC; Sigma-Aldrich), and digested with Sequencing Grade Modified Trypsin (Promega) in approximate ratio of 1:50 to 1:20 (w/w) at 37 °C for 16 h. Finally, the peptide samples were desalted with C18 Macrospin columns (Nest Group). Desalted peptides were divided for whole proteome analysis and the following phosphoproteome analysis. For phosphopeptide enrichment, desalted peptide mixtures were firstly loaded into a stage-tip packed with Ti_4_-IMAC microspheres which were prewashed with loading buffer (80% acetonitrile/6% trifluoroacetic acid (TFA)). The Ti_4_-IMAC microspheres with enriched phosphopeptides were collected by centrifugation. To remove nonspecifically absorbed peptides, the Ti_4_-IMAC microspheres were washed with 50% acetonitrile/6% TFA/200 mM NaCl and 50% acetonitrile/0.1% TFA sequentially. To elute the enriched phosphopeptides, elution buffer containing 10% NH_4_OH was added and the enriched phosphopeptides were eluted with centrifugation. The supernatant containing phosphopeptides was collected and lyophilized for LC-MS/MS analysis.

### Proximity labeling

For BioID-MS approach, cell pellets were thawed in 3 mL of ice-cold lysis buffer (0.5% IGEPAL, 50 mM HEPES, pH 8.0, 150 mM NaCl, 50 mM NaF, 1.5 mM NaVO_3_, 5 mM EDTA, 0.1% SDS, 0.5 mM PMSF, and protease inhibitors), and lysates were sonicated and treated with Benzonase® Nuclease (Santa Cruz Biotechnology, sc-202391). Lysate was then centrifuged, and clear solution containing target proteins was obtained. Lysate was subjected to a one-step purification via Strep-Tactin® Sepharose® resin (IBA). A detailed description of the method used here can be found in a previous protocol^57^.

### MS sample preparation

Purified protein lysis sample was prepared further for LC-MS. To break disulfide bonds, the samples were incubated with 5 mM TCEP (Sigma-Aldrich, #C4706, USA) at 37 °C for 20 min. Samples were let cool to room temperature and alkylated with 10 mM iodoacetamide (Fluka, Sigma-Aldrich, #57670, USA) at room temperature for 20 min. Samples were trypsin-digested with 1.5 µg Sequencing Grade Modified Trypsin (V5111, Promega) at 37 °C for 16 h. After digestion peptides were quenched with 10% TFA and finally, desalted with BioPureSPN MINI C18 columns (The nest Group, Inc.). A detailed description of the method used here can be found in a previous protocol^57^. The dried peptides were reconstituted in 30 µL Buffer A (0.1% (vol/vol) TFA and 1% (vol/vol) acetonitrile in HPLC water). Samples were further diluted 1 + 19 µL with HPLC water containing 0.1% (vol/vol) formic acid. The manufacturer’s instructions were followed to load into Evotips (Evosep).

### LC-MS analysis

The desalted samples underwent analysis using the Evosep One liquid chromatography system, which was coupled to a hybrid trapped ion mobility quadrupole TOF mass spectrometer (Bruker timsTOF Pro) through a CaptiveSpray nano-electrospray ion source. Peptide separation was achieved using an 8 cm × 150 µm column packed with 1.5 µm C18 beads (EV1109, Evosep) following the 60-sample-per-day method, which employed a 21-min gradient time. Mobile phases A and B consisted of 0.1% formic acid in water and 0.1% formic acid in acetonitrile, respectively. For BioID samples, the MS analysis was conducted in positive-ion mode using a data-dependent acquisition (DDA) approach in PASEF (Parallel Accumulation Serial Fragmentation) mode, utilizing the DDA-PASEF-short_gradient_0.5s-cycletime method. The DIA MS analysis was performed in the positive-ion mode with DIA-PASEF method^105^ with sample optimized DIA scan parameters. We performed DDA in PASEF mode from a pooled sample to be able to adjust DIA-PASEF parameters optimally to each sample type. To perform sample-specific DIA-PASEF parameter adjustment, the default DIA-short-gradient acquisition methods were adjusted based on the sample-specific DDA-PASEF run with the software ‘tims Control’ (Bruker Daltonics). The following parameters were modified for each sample type: phoshory enrichment, mass range 433–1684 Da, mobility range 0.85–1.3 1/K0, cycle time estimate 1.80 s and for total proteome, mass range 370–1370 Da, mobility range 0.85–1.30 1/K0, with cycle time estimate 1.27 s. The ion mobility windows were set to best match the ion cloud density from the sample type-specific DDA-runs.

Following the analysis, the raw data obtained for both total proteome and phosphoproteomics underwent processing using DIA-NN (version 1.8.1) software. For total proteome DIA-NN, the parameters included a maximum of 1 missed cleavage, up to 3 variable modifications, N-terminal methionine excision, carbamidomethylation of cysteine residues, oxidation of methionine, acetylation of the N-terminus, peptide length ranging from 7 to 30 amino acids, precursor charge ranging from 2 to 4, precursor *m/z* range of 300–1600, fragment ion *m/z* range of 100–1700, and mass accuracy of 15 ppm, with MS1 accuracy also set at 15 ppm. For phosphoproteomics DIA-NN, the parameters were similar, with 1 missed cleavage allowed, a maximum of 3 variable modifications, N-terminal methionine excision, carbamidomethylation of cysteine residues, exclusion of oxidation on methionine residues, acetylation permitted at the N-terminus, peptide length ranging from 7 to 30 amino acids, precursor charge ranging from 2 to 4, precursor *m/z* range of 300–1600, fragment ion *m/z* range of 100–1700, and a mass accuracy of 15 ppm, with MS1 accuracy also set at 15 ppm. BioID raw data were processed with FragPipe v17.1 utilizing MSFragger^106^ against reviewed human entries of the UniProtKB database (downloaded 8.3.2022) coupled with SARS-CoV-2 protein information derived from GISAID. Carbamidomethylation of cysteine residues was used as the static modification. N-terminal acetylation and oxidation of methionine were used as the dynamic modification. Biotinylation of lysine and N-termini were set as variable modifications. Trypsin was selected as the enzyme, and maximum of two missed cleavages were allowed. Both instrument and label-free quantification parameters were left at default settings. Final results from these steps are Spectral Counts values from peptides with FDR < 0.01 from Philosopher.

### Identification of high-confidence PPIs

The web tool (http://proteomics.fi/) incorporated with Significance Analysis of INTeractome (SAINT)-express version 3.6.3^107^ was used as a statistical approach for identification of specific HCIs from BioID-MS data. HCIs were defined by an estimated protein-level Bayesian FDR (BFDR) of ≤ 0.05. Furthermore, we used our own in-house contaminant GFP library for Proximity Dependent Biotinylation with a cutoff frequency of ≥ 50%, except for an average spectral count fold change ≥ 3 to remove non-specific interactors. Most common MS contaminants were removed from results. Results are bait normalized.

### MS-microscopy analyses

For any bait of interest, the averaged peptide-spectrum match values of each prey protein were calculated and uploaded to the web tool (http://proteomics.fi/) to calculate their subcellular distribution.

### Immunostaining and fluorescence microscopy

Transfected U2OS U2-OS (ATCC®, HTB-96™) cells were cultivated in 96-well optical bottom plate (Brooks, MGB096-1-2-LG-L). After fixation in 4% paraformaldehyde in PBS and permeabilization in Triton X-100, cells were incubated with primary (anti-HA IgG1, BioLegends, 901502) antibodies. Next, cells were washed twice with PBS and incubated with PhenoVue Fluor 488-Donkey Anti-Mouse Antibody Cross-Adsorbed (PerkinElmer, 2DXM488C1), PhenoVue Fluor 568-Phalloidin (PerkinElmer, CP25681), PhenoVue Fluor 647-Concanavalin A (PerkinElmer, CP96471), DAPI (Santa Cruz, 28718-90-3). After staining, cells were washed twice and imaged in PBS. Imaging was performed using a Molecular Devices Nano high-content screening microscope, using Nikon 20×/0.45 S Plan Fluor ELWD, WD 8.2–6.9 mm (pixel size of 0.328 μm) objective. The following filter sets were used for multicolor acquisition: DAPI (Ex: 377/50 nm, Em: 447/60 nm, Dichroic: 409 nm), GFP (Ex: 472/30 nm, Em: 520/35 nm, Dichroic: 495 nm), Texas Red (Ex: 562/40 nm, Em: 624/40 nm, Dichroic: 593 nm), Cy5 (Ex: 628/40 nm, Em: 692/40 nm, Dichroic: 660 nm). Images were stored as TIFF and processed (linear intensity scaling, pseudocoloring, composite panel assembly) using a custom-made python script.

### Co-IP

For co-IP validation, HEK293 cells (5 × 10^5^ per well) were seeded in a 6-well plate and co-transfected with 500 ng each of Strep-HA-tagged prey and V5-tagged bait constructs using Fugene 6. After 24 h, cells were washed with ice-cold PBS and lysed with 1 mL of HENN lysis buffer (50 mM HEPES, pH 8.0, 5 mM EDTA, 150 mM NaCl, 50 mM NaF, 0.5% IGEPAL, 1 mM DTT, 1 mM PMSF, 1.5 mM Na_3_VO_4_, and 1× protease inhibitor) on ice. Lysates were centrifuged at 16,000 × *g* for 20 min at 4 °C to obtain the supernatant.

In parallel, 30 µL of Strep-Tactin Sepharose resin was washed twice with 200 µL of HENN lysis buffer. The clear lysate was then incubated with the beads for 1 h at 4 °C on a rotation wheel. After incubation, the beads were collected by centrifugation, washed three times with 1 mL of HENN buffer, and each wash involved centrifugation at 4000 × *g* for 30 s at 4 °C. Finally, 60 µL of 2× Laemmli buffer was added to the beads, boiled at 95 °C for 5 min, and the samples were analyzed via dot blot to validate PPIs.

For dot blot analysis, the Bio-Dot Microfiltration system was used as per the manufacturer’s instructions. The nitrocellulose membrane was pre-washed with TBS, and 10 µL of the sample was spotted and drained under vacuum. The membrane was blocked with 5% milk in TBS-T for 60 min, followed by incubation with mouse anti-V5 (1:5000) overnight at 4 °C. After washing with TBS-T, HRP-conjugated goat anti-mouse (1:2000) was applied for 60 min at room temperature. The membrane was washed, incubated with ECL solution, and imaged.

To detect additional targets, the membrane was stripped, re-blocked, and incubated with rabbit anti-HA (1:2000) overnight at 4 °C. After washing, goat anti-rabbit (1:2000) was applied, and the membrane was re-imaged to detect HA-tagged proteins, confirming the protein interaction.

### Data preprocessing

The experimental data underwent bait normalization as a preliminary step. Following bait normalization, missing values in the data were imputed using the QRILC (Quantile Regression-based Imputation using Locally Constant functions) method using the ‘imputeLCMD’ R package and R (version 4.3.1). Furthermore, median normalization was conducted on the data as a normalization step prior to analysis.

### Correlation analysis

A differential abundance analysis was performed to compare protein abundance levels across sample groups. Log_2_FC and *P*-value were calculated using python (version 3.9.7) and ttest_ind from scipy.stats python package based on the imputed and normalized intensity data. The *P*-values were corrected using Benjamini-Hochberg correction method from the ‘multitest’ module of the ‘statsmodels.stats’ package. Figures were generated using plotly python library.

### GO enrichment analysis

We employed the DAVID tool to unravel the functional intricacies of the proteome dataset. The primary goal was to uncover enriched GO terms and pathways that shed light on the molecular context of the identified proteins. Our investigation encompassed three key aspects of GO: Biological Process (BP), Cellular Component (CC), and Molecular Function (MF). DAVID discerned GO terms that exhibited significant enrichment within our protein dataset. Simultaneously, we analyzed cellular pathways by applying the KEGG and Reactome pathway analyses^108,109^.

### Structural analysis of mutation sites

In order to present the three-dimensional (3D) structure of our proteins of interest, PyMol version 2.5 (Schrödinger LLC, New York, NY, USA) was used. The crystal structures of the SARS-CoV-2 proteins were obtained from the Protein Data Bank (PDB)^110^, while de novo models were taken from the AlphaFold protein folding prediction database and Swiss-Prot database. Figures of the protein structure were generated using the PyMol software^111,112^.

### In silico protein structure preparation

For each protein of interest, AlphaFold models (v4) were collected from the AlphaFold Protein Structure Database^111^, https://alphafold.ebi.ac.uk (accessed August 14th 2024). All available X-ray diffraction and EM structures were collected from the RCSB protein databank^110^, https://www.rcsb.org/ (accessed August 14th 2024). Experimental structures were preprocessed with an in-house workflow using Python 3.12 and BioPython 1.83 as follows: Structures with less than 80 amino acid length were dropped. Relevant chains were extracted from multi-protein complexes. Where possible, the preferred biological assembly as recorded in the RCSB PDB was retained for homo-multimeric structures, with monomers serving as a fallback. All water molecules, non-structural ions, solvents and ligands except cofactors (heme groups, iron-sulfur clusters and hybrids, NADH, NADPH, FAD and FMN) were removed from the structures.

All structures were prepared with the Schrödinger Protein Preparation Wizard^113,114^ using default settings. Hydrogens were added, relevant bond orders and cofactor charge states assigned, where applicable. The protein amino acid protonation states were determined with PROPKA (pH 7) prior to hydrogen bonding network optimization. All structures were subjected to a restrained minimization in the OPLS4 force field using standard settings.

### Pocket druggability calculations

Schrödinger SiteMap was used for site prediction and druggability analyses^114–116^. All targets were screened for well-defined druggable sites using SiteMap with standard settings. Sites with a druggability score (DScore) of 0.8 or higher were considered druggable^115^. To detect more shallow, hydrophobic sites often associated with PPIs, a modified SiteMap protocol described by Loving et al. 2014 was used^60^. Modified SiteMap parameters were employed as previously described (grid = 0.35), modphobic = 0, maxdist = 10, enclosure = 0.4, maxvdw = 1.0, dtresh = 5.0, mingroup = 7 and nthresh = 7^60^ and the modified druggability descriptor DScore+ was computed (DScore + 0.3 * hydrophobic). Candidate sites with volumes of 160–800 Å^3^ were labeled druggable PPI sites. Larger pocket regions were counted separately as ‘large PPI sites’. Pockets with a volume below 160 Å^3^ were considered cryptic. Cryptic pockets with DScore+ of at least 1.3 were subjected to a two-step induced fit docking (IFD) pipeline using Schrödinger Glide and Prime^114,117^ as previously described^60^. Briefly, grid centers were determined as centroids of all site residues. IFD parameters were kept at defaults except for the outer box size being set to 25 Å. In the first step, a naphthalene molecule was docked to the site and, if poses were retrieved, the two top-scoring complexes by IFDscore were progressed into the second stage. Using the same grid center as in stage 1 and removing the naphthalene ligand from the receptor structure, the tetra-substituted naphthalene derivative was docked in another IFD run^60^. All resulting complexes from stage 2 were subjected to SiteMap analysis with the parameter set for PPI sites and evaluating only the site with the bound naphthalene derivative. Cryptic sites with a DScore+ of at least 1.7 and a volume of at least 160 Å^3^ were labeled druggable cryptic sites. Smaller sites matching the DScore+ requirement were recorded with the keyword cryptic, but not considered druggable.

Since both the original and the modified SiteMap protocol were validated on X-ray crystallographic structures, EM structures with their often significantly lower resolution and AlphaFold models with their potential structural uncertainties were analyzed separately in all steps.

### Assessment of known ligand profiles

SMILES for all ligands in the experimental structures were collected via the RCSB GraphQL API with an in-house Python 3.12 workflow and standardized and analyzed with RDKit v2023.09.4. Molecular weight (MW), heavy atom count, numbers of hydrogen-bond (HB) donors and acceptors and log*P* were computed and ligands were classified as follows: ‘Extended drug-like’: MW 200–650 Da, at least 10 heavy atoms; ‘Satisfies Rule-of-Five (ROF)’: MW < 500 Da, HB donors ≤ 5, HB acceptors ≤ 10, log*P* < 5;‘iPPI-like violations’: up to two violations to ROF MW ≥ 500 Da and log*P* > 5 were allowed^61^.

### Assessment of literature coverage and target status

Each protein of interest was queried in PubMed with the Entrez API of BioPython 1.83. Total counts returned for each query were counted and no further processing or curation was done. Each query was run once for each protein of interest, including the protein name as obtained from UniProt (The UniProt Consortium 2023). All terms were searched across all available fields and by MeSH terms. The following keywords were used: (i) ‘drug design’ or ‘drug discovery’, ‘Covid-19’ or ‘SARS-CoV-2’ and ‘protein expression’ and ‘assay’ to identify potential coverage in SARS-CoV-2 literature with experimental assay on protein level; (ii) ‘drug design’ or ‘drug discovery’, ‘Covid-19’ or ‘SARS-CoV-2’ to assess any literature on the target candidate in the context of SARS-CoV-2; and (iii) ‘drug design’ or ‘drug discovery’ and ‘protein expression’ and ‘assay’ to determine whether the target candidate was previously studied (experimentally) in any other context. Full queries are available as Supplementary information.

Additionally, UniProt accession codes were mapped against targets in the Therapeutic Target Database (TTD, https://db.idrblab.net/ttd (accessed September 15th 2024))^63^ and target classes for all targets in the TTD were recorded.

### Classification by druggability confidence

To indicate the confidence in the druggability of a protein of interest, all proteins were grouped in druggability categories based on the analysis results, ranging from most likely druggable (4) to not druggable (0). Since SiteMap had not been validated with EM structures or AlphaFold models, druggability indicated by their analysis was considered ‘likely druggable’ (3). When different structural datasets indicated different druggability, but the majority suggested druggable sites, we classified the target as ‘potentially druggable’ (2) or ‘possibly not druggable’ (1), if the majority suggested no druggable sites. In the analysis of known ligands, the presence of only iPPI-like ligands was considered ‘potentially druggable’ (2) since RoF violations can generally be associated with a loss of (orally available) drug-likeness and may occur in compounds that are not iPPIs. For literature and TTD-analysis, clinical, patented and established targets are flagged as ‘druggable’, research-level targets (in TTD or at least 100 PubMed results) as ‘potentially druggable’, and cases where at least 10 PubMed results were retrieved were assigned 1 as ‘potential research targets’. All scores from individual groupings were summed up into a druggability confidence score to reflect the amount of evidence that the protein in question is druggable, with higher confidence scores being more likely druggable.

## Supplementary information

Supplementary Table S1

Supplementary Table S2

Supplementary Table S3

Supplementary Table S4

Supplementary Table S5

Supplementary Table S6

Supplementary Table S7

Supplementary Table S8

Supplementary Table S9

Supplementary Figures

## Data Availability

Plasmids used can be found in Addgene (https://www.addgene.org/Markku_Varjosalo/). The datasets generated and analyzed during the current study are available in the massive database repository, https://massive.ucsd.edu/ProteoSAFe/static/massive.jsp, under accession code MSV000096339.
