# Supplementary material for: The comprehensive SARS-CoV-2 ‘hijackome’ knowledge base
Source: Cell Discov. 2024 Dec 9;10:125. doi: 10.1038/s41421-024-00748-y (PMC11628605; doi:10.1038/s41421-024-00748-y)
Supplement: Supplementary file 10 — Supplementary Figures [file 41421_2024_748_MOESM10_ESM.pdf]

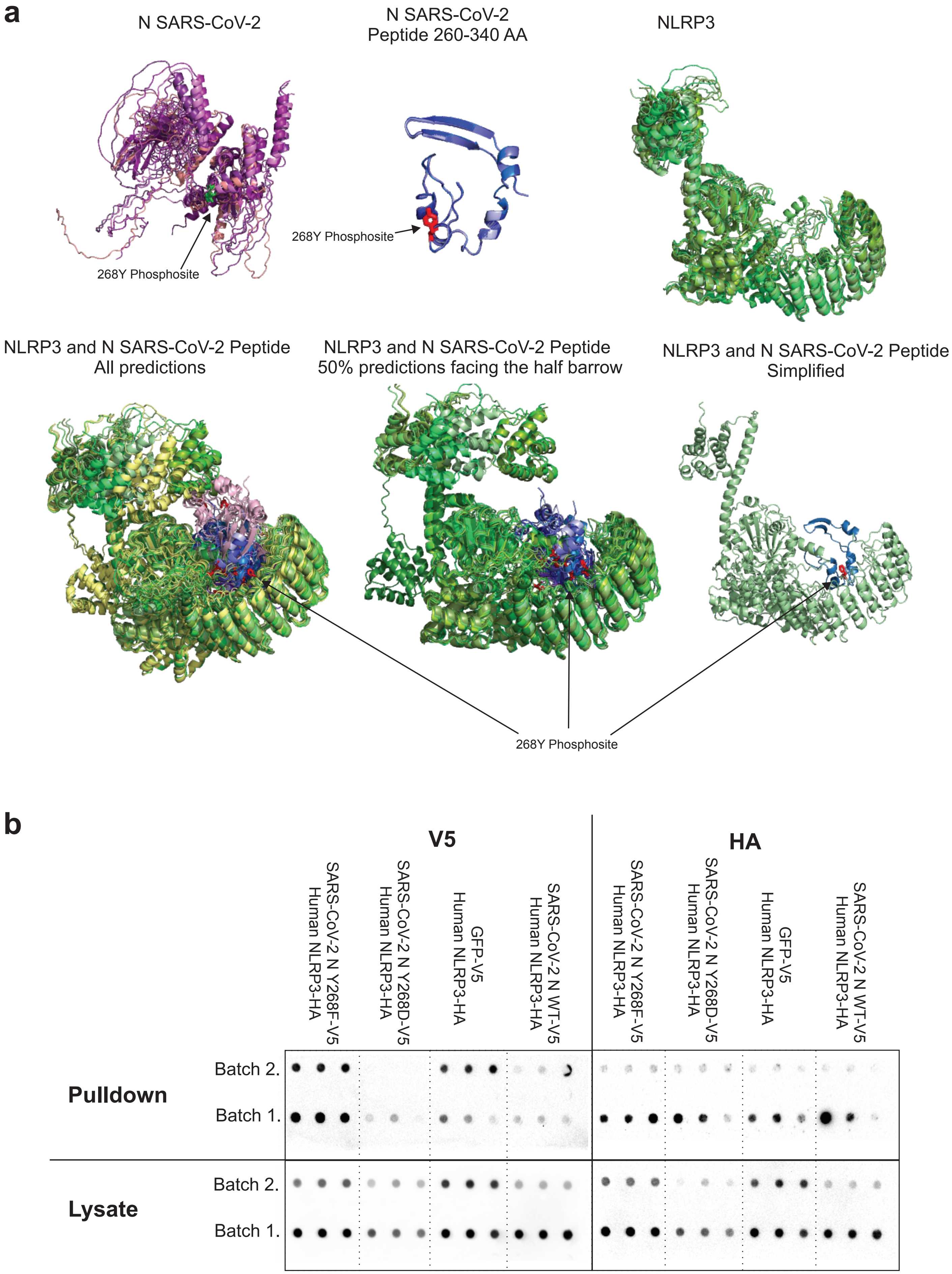

a
N SARS-CoV-2
N SARS-CoV-2
Peptide 260-340 AA
NLRP3
268Y Phosphosite
268Y Phosphosite
NLRP3 and N SARS-CoV-2 Peptide
All predictions
NLRP3 and N SARS-CoV-2 Peptide
50% predictions facing the half barrow
NLRP3 and N SARS-CoV-2 Peptide
Simplified
268Y Phosphosite
b
V5
HA
SARS-CoV-2 N Y268F-V5
Human NLRP3-HA
SARS-CoV-2 N Y268D-V5
Human NLRP3-HA
GFP-V5
Human NLRP3-HA
SARS-CoV-2 N WT-V5
Human NLRP3-HA
SARS-CoV-2 N Y268F-V5
Human NLRP3-HA
SARS-CoV-2 N Y268D-V5
Human NLRP3-HA
GFP-V5
Human NLRP3-HA
SARS-CoV-2 N WT-V5
Human NLRP3-HA
Pulldown
Batch 2.
Batch 1.
Lysate
Batch 2.
Batch 1.

Supplementary Fig S1. Structural Interaction and Co-Immunoprecipitation Analysis of NLRP3 with SARS-CoV-2 Nucleocapsid Protein. **a)** Structural Predictions of NLRP3 and SARS-CoV-2 N Protein by AlphaFold. In the first row, from left to right, we present the following: an overlay of N SARS-CoV-2 AlphaFold 3 predictions in purple with the 268Y phosphosite localization shown in green; an overlay of N SARS-CoV-2 peptide (260-340 AA) AlphaFold 3 predictions with the 268Y phosphosite shown in red; and an overlay of NLRP3 AlphaFold predictions in green. In the second row, we display NLRP3 and N SARS-CoV-2 peptide with all predictions from AlphaFold 3. Yellow/pink indicates non-uniform structural predictions, usually with the 268Y phosphosite facing outside of the NLRP3 half-barrel. Green (NLRP3) and blue (N peptide) show predictions with the 268Y phosphosite facing the NLRP3 half-barrel. The 268Y phosphosite is indicated in red. Next, we show predictions where the 268Y phosphosite is facing the NLRP3 half-barrel, using the same color coding as in the previous image. Lastly, we present a simplified image of the NLRP3 and N peptide, using the same color scheme as in the last two images. **b)** co-immunoprecipitation experiments that assess the physical interaction between the SARS-CoV-2 N protein and NLRP3. The "Beads" and “Lysate” section shows the outcome of two experimental batches, with protein detection carried out using either the V5 or HA antibody. Various constructs of the SARS-CoV-2 N protein, including the wild-type and Y268F mutant, were tested alongside human NLRP3 tagged with HA. The "Lysate" section serves as a control, confirming the expression of these constructs in the cell lysates prior to immunoprecipitation.

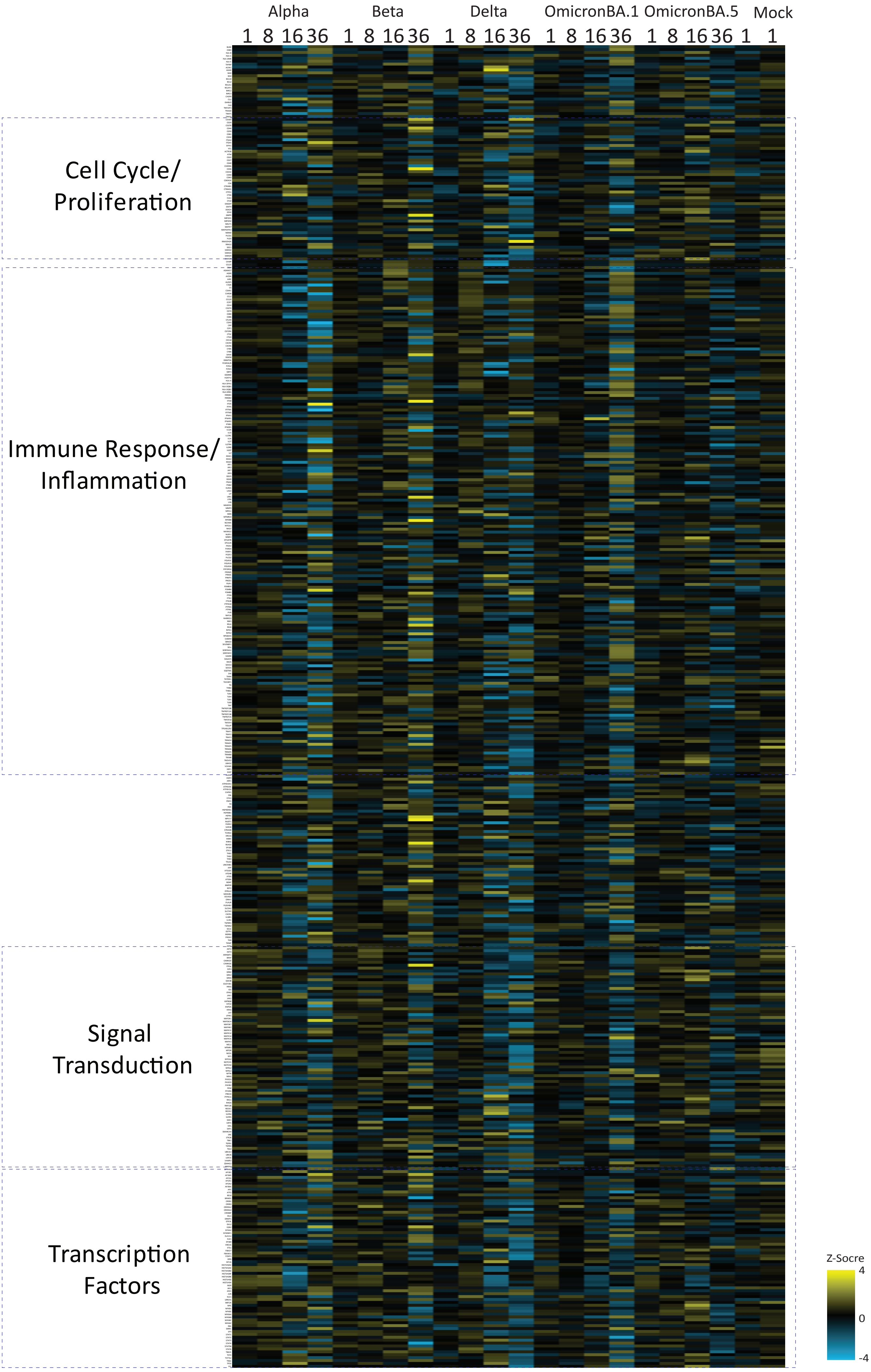
Alpha
Beta
Delta
OmicronBA.1
OmicronBA.5
Mock
1 8 16 36 1 8 16 36 1 8 16 36 1 8 16 36 1 8 16 36 1 1
Cell Cycle/ Proliferation
Immune Response/ Inflammation
Signal Transduction
Transcription Factors
Z-Socre
4
0
-4

Supplementary Fig S2. NanoString analysis. Transcriptome expression analysis via the NanoString RNA analysis of the SARS-CoV-2 Variants (Alpha, Beta, Delta, Omicron BA.1, and Omicron BA.5) in various hours-post infection (hpi). 457 genes were identified across the analyzed samples. The genes are groups based on their molecular functions. The color gradient shows the Z-score (-4 to +4).

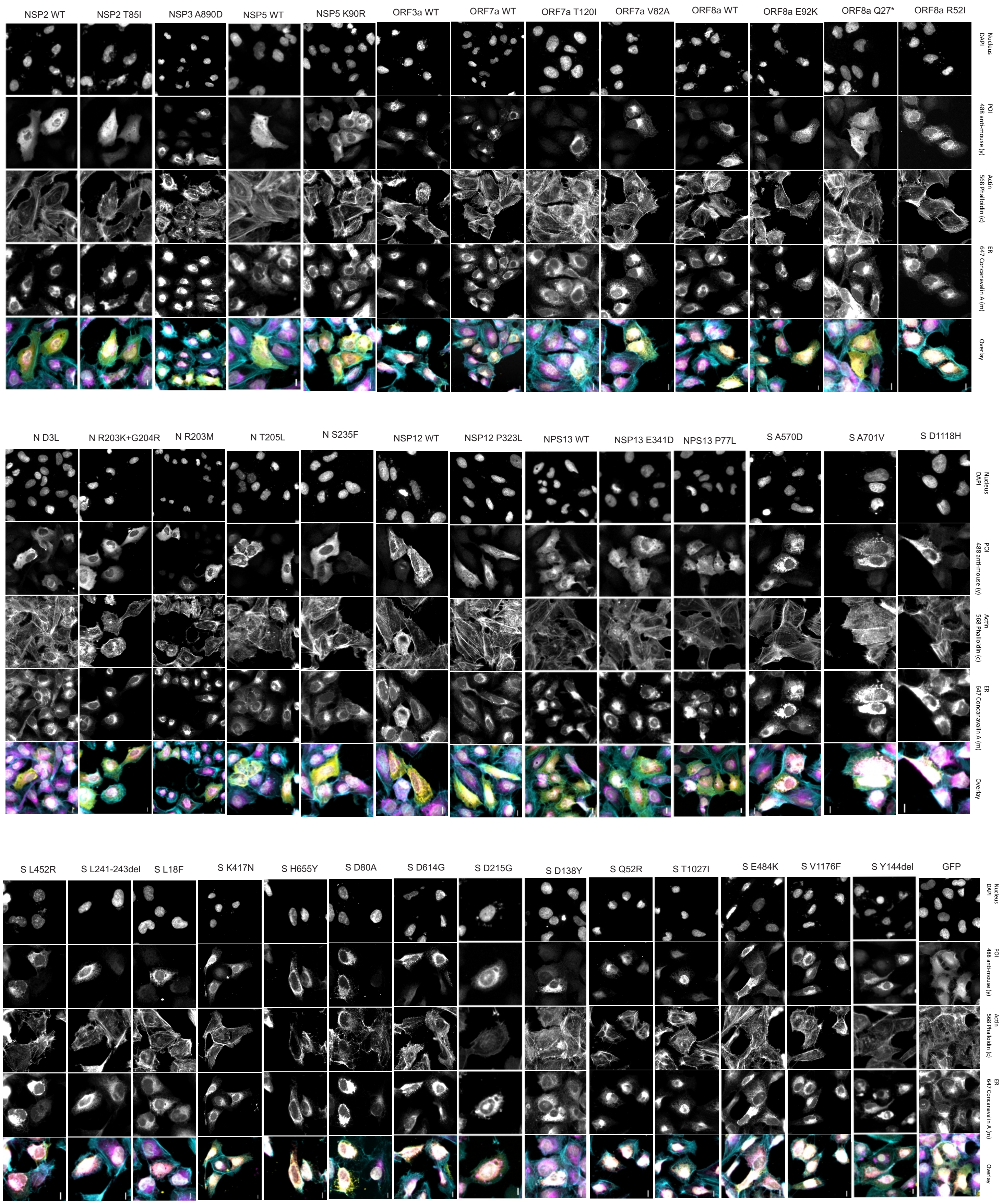

Scale bar, 10 microns (10/0.335=30pxs)

Supplementary Fig S3. **Remaining constructs** of **molecular microscopy and immunofluorescence analysis of the SARS-CoV-2 mutations.** Immunofluorescence microscopy images of all other SARS-CoV-2 proteins expressed from the MAC-tagged viral ORFs, transfected into U2-OS cells, and detected by immunofluorescence. Scale bar is 10 µm.

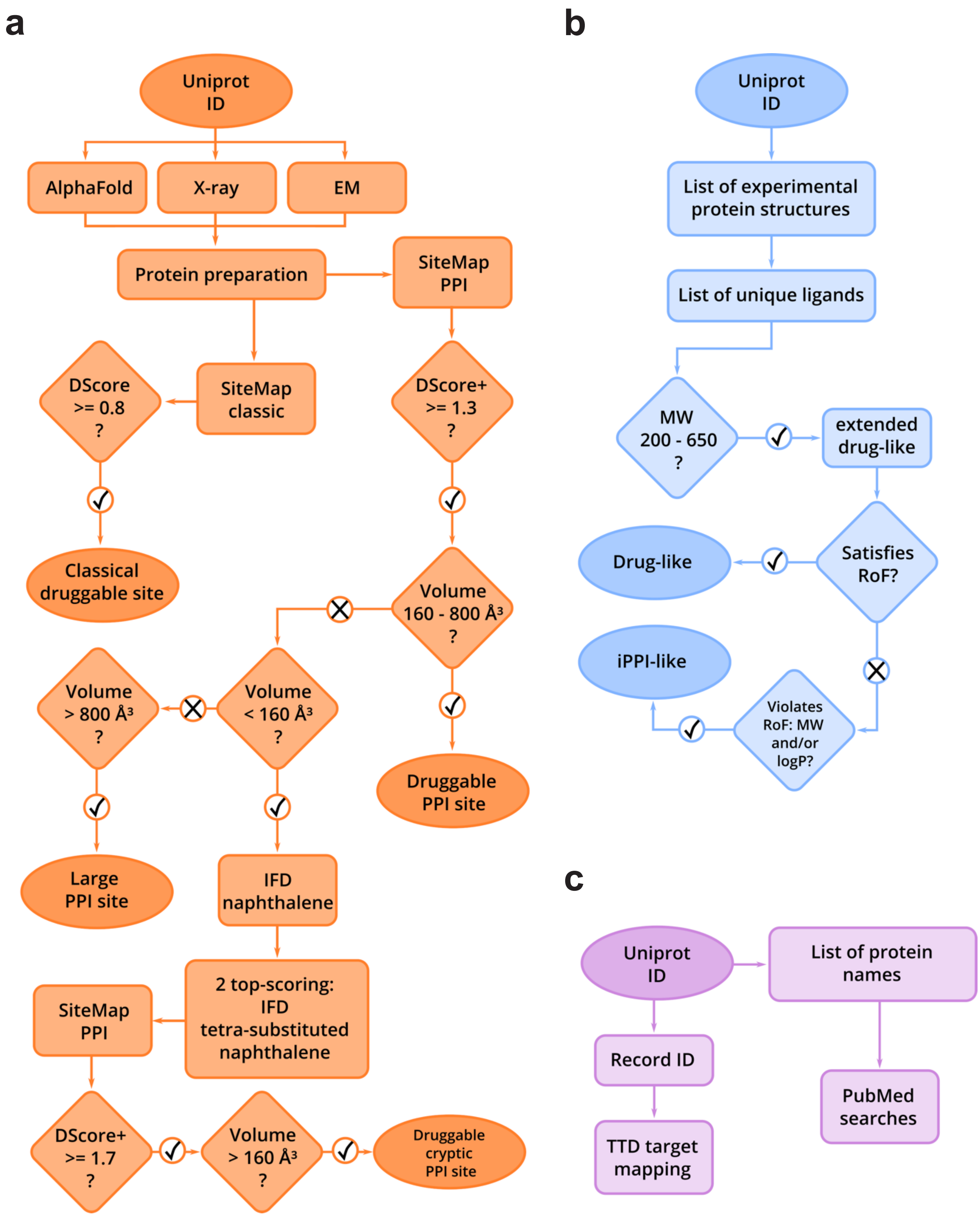
a
Uniprot ID
AlphaFold
X-ray
EM
Protein preparation
SiteMap PPI
SiteMap classic
DScore >= 0.8 ?
Classical druggable site
DScore+ >= 1.3 ?
Volume 160 - 800 Å³ ?
Druggable PPI site
Volume < 160 Å³ ?
Volume > 800 Å³ ?
Large PPI site
IFD naphthalene
2 top-scoring: IFD tetra-substituted naphthalene
SiteMap PPI
DScore+ >= 1.7 ?
Volume > 160 Å³ ?
Druggable cryptic PPI site
b
Uniprot ID
List of experimental protein structures
List of unique ligands
MW 200 - 650 ?
extended drug-like
Satisfies RoF?
Drug-like
iPPI-like
Violates RoF: MW and/or logP?
c
Uniprot ID
List of protein names
Record ID
TTD target mapping
PubMed searches

Supplementary Fig S4. Schematic illustrations of workflows for target druggability assessment. a) Structure-based pocket prediction and druggability analysis workflow with Schrödinger SiteMap and induced fit docking (IFD) steps to open cryptic pockets with limited protein flexibility modeling. PPI = protein:protein interaction. **b**) Evaluation of properties of ligands in experimental target structures. RoF = Rule-of-Five; MW = molecular weight; iPPI = PPI inhibitor. **c**) Workflow to assess literature coverage by PubMed searches and cross-analysis with the Therapeutic Target Database (TTD).
